# Asymmetry analysis of nuclear Overhauser enhancement effect at ‐1.6 ppm in ischemic stroke

**DOI:** 10.1002/mp.17677

**Published:** 2025-02-11

**Authors:** Yu Zhao, Aqeela Afzal, Zhongliang Zu

**Affiliations:** ^1^ Vanderbilt University Institute of Imaging Science Vanderbilt University Medical Center Nashville USA; ^2^ Department of Radiology and Radiological Sciences Vanderbilt University Medical Center Nashville USA; ^3^ Department of Radiology and Functional and Molecular Imaging Key Laboratory of Sichuan Province West China Hospital of Sichuan University Chengdu China; ^4^ Department of Neurological Surgery Vanderbilt University Medical Center Nashville USA; ^5^ Department of Biomedical Engineering Vanderbilt University Nashville USA

**Keywords:** chemical exchange saturation transfer (CEST), ischemic stroke, nuclear Overhauser enhancement (NOE)

## Abstract

**Background:**

The nuclear Overhauser enhancement (NOE)‐mediated saturation transfer effect at ‐1.6 ppm, termed NOE(‐1.6 ppm), has demonstrated potential for detecting ischemic stroke. However, the quantification of the NOE(‐1.6 ppm) effect usually relies on a multiple‐pool Lorentzian fit method, which necessitates a time‐consuming acquisition of the entire chemical exchange saturation transfer (CEST) Z‐spectrum with high‐frequency resolution, thus hindering its clinical applications.

**Purpose:**

This study aims to assess the feasibility of employing asymmetry analysis, a rapid CEST data acquisition and analysis method, for quantifying the NOE(‐1.6 ppm) effect in an animal model of ischemic stroke.

**Methods:**

We examined potential contaminations from guanidinium/amine CEST, NOE(‐3.5 ppm), and asymmetric magnetization transfer (MT) effects, which could reduce the specificity of the asymmetry analysis of NOE(‐1.6 ppm). First, a Lorentzian difference (LD) analysis was used to mitigate direct water saturation and MT effects, providing separate estimations of the contributions from the guanidinium/amine CEST and NOE effects. Then, the asymmetry analysis of the LD fitted spectrum was compared with the asymmetry analysis of the raw CEST Z‐spectrum to evaluate the contribution of the asymmetric MT effect at ‐1.6 ppm.

**Results:**

Results show that the variations of the LD quantified NOE(‐1.6 ppm) in stroke lesions are much greater than that of the CEST signals at +1.6 ppm and NOE(‐3.5 ppm), suggesting that NOE(‐1.6 ppm) has a dominating contribution to the asymmetry analysis at ‐1.6 ppm compared with the guanidinium/amine CEST and NOE(‐3.5 ppm) in ischemic stroke. The NOE(‐1.6 ppm) variations in the asymmetry analysis of the raw CEST Z‐spectrum are close to those in the asymmetry analysis of the LD fitted spectrum, revealing that the NOE(‐1.6 ppm) dominates over the asymmetric MT effects.

**Conclusion:**

Our study demonstrates that the asymmetry analysis can quantify the NOE(‐1.6 ppm) contrast in ischemic stroke with high specificity, thus presenting a viable alternative for rapid mapping of ischemic stroke.

## 1 INTRODUCTION

The emerging MR molecular imaging technique, chemical exchange saturation transfer (CEST) MRI, enables highly sensitive detection of target molecules and their chemical environment in biological tissues.^1^, ^2^, ^3^, ^4^, ^5^ In CEST imaging, a Z‐spectrum, which represents the water signal as a function of the frequency offset (Δω) of the saturation pulses, allows us to identify molecules with specific resonance frequency offsets. A number of CEST effects have been reported to carry useful information for the diagnosis of diseases. These effects include amide proton transfer (APT) at approximately 3.5 ppm,^6^ guanidinium (guan) CEST at approximately 2 ppm,^7^, ^8^ amine CEST at approximately 3 ppm,^9^, ^10^ nuclear Overhauser enhancement (NOE)‐mediated saturation transfer effects at approximately ‐1.6 ppm, termed NOE(‐1.6 ppm),^11^, ^12^ and at approximately ‐3.5 ppm,^13^, ^14^, ^15^ termed NOE(‐3.5 ppm). However, it is important to note that the direct water saturation (DS) and magnetization transfer (MT) effects often overlap with the CEST/NOE effects. In addition, water longitudinal relaxation time (T_1w _= 1/R_1w_) influences all CEST signals.^16^, ^17^ These overlapping components and T_1w_ always vary in diseases, complicating the interpretation of variations of the CEST/NOE effects. Thus, a careful analysis is required to quantify the individual contribution of a certain CEST effect.

In recent years, the applications of CEST/NOE effects in detecting ischemic stroke have been widely studied. The APT^6^, ^18^, ^19^, ^20^, ^21^, ^22^, ^23^, ^24^, ^25^, ^26^, ^27^, ^28^, ^29^ and guanidinium CEST^22^, ^30^, ^31^ have shown obvious variations in ischemic stroke, providing informative image contrasts for probing tissue acidification in CEST imaging. The amine CEST effect may also vary in ischemic stroke.^32^ Furthermore, the NOE(‐3.5 ppm) effect has been observed to change in ischemic stroke, which is considered as a response to pH variations.^33^ Note that other studies reported that the NOE(‐3.5 ppm) is insensitive to pH variations during ischemic stroke.^34^, ^35^ Additionally, our previous study showed that the NOE(‐1.6 ppm) effect could also be utilized for detecting ischemic stroke.^36^ Previously, we have also demonstrated that the NOE (‐1.6 ppm) effect arises from membrane choline phospholipids.^12^ Thus, the variation of the NOE (‐1.6 ppm) effect in ischemic stroke may reflect changes in membrane properties, which is different from the origin of other CEST/NOE effects in ischemic stroke. The NOE(‐1.6 ppm) imaging based on this mechanism has the potential to enhance clinical diagnosis and contribute to a better understanding of the physiological and pathological changes in brain tissue following a stroke.

Despite the potential of CEST/NOE effects in ischemic stroke mapping, the intricate interplay between these effects, as well as the background DS and MT effects, poses challenges in interpreting contrasts observed in CEST/NOE images. Thus, quantifying these CEST/NOE effects and improving their specificity are critical for their applications. Various multiple‐pool Lorentzian fit methods have been used to quantify CEST effects. However, these methods, which require the acquisition of the entire or a large portion of the Z‐spectrum with numerous frequency offsets, are time‐consuming. In addition, the fitting method is not robust when the signal‐to‐noise ratio (SNR) of CEST signals is low. These limitations make them impractical for clinical stroke imaging, and thus developing fast and robust imaging methods is imperative. To remove the confounding factors of DS and MT effects, an asymmetry analysis of magnetization transfer ratio (MTR_asym_), which could be conducted with a sparse CEST Z‐spectrum acquisition, has been widely employed in APT‐weighted imaging. This method involves subtracting CEST signals acquired symmetrically around the center of the water resonant peak to eliminate DS and MT effects that are assumed to be symmetric in many analyses. However, it is important to note that the subtraction method introduces additional contaminations from the NOE(‐3.5 ppm) and asymmetric MT effect,^37^ as previously described by,^1^

(1) $${\text{MTR}}_{\text{asym}}\left(\Delta \omega \right)=\text{APTR}\left(\Delta \omega \right)+{\text{MTR}}_{\text{asym}}^{\prime }\left(\Delta \omega \right)$$

where APTR is the amide proton transfer ratio, which represents the contribution from only the APT effect; ${\text{MTR}}_{\text{asym}}^{\prime }$ represents the non‐specific contributors from the NOE(‐3.5 ppm) and the asymmetric MT effect.

The MTR_asym_ metric, while not specific to APTR due to the presence of ${\text{MTR}}_{\text{asym}}^{\prime }$, can still be useful in some situations where the variations of ${\text{MTR}}_{\text{asym}}^{\prime }$ in specific pathologies are relatively small and can be neglected. In such cases, the asymmetry analysis method can effectively reflect the variations of APTR in pathologies. Moreover, the asymmetry analysis method is relatively easy to perform, model‐free, and significantly faster compared to other fitting methods, making it a widely adopted technique for APT‐weighted imaging in practice.^6^, ^38^

When applying the asymmetry analysis method to extract the NOE(‐1.6 ppm) effect, it is important to consider potential contributions from the pools near ± 1.6 ppm. These confounding effects include the guanidinium CEST effect at approximately 2 ppm and the asymmetric MT effect centered at approximately ‐2.3 ppm,^37^ which may contribute to the signal variations at ± 1.6 ppm. In addition, the amine CEST effect at 3 ppm and NOE(‐3.5 ppm) effect have broad peaks in Z‐spectrum, which may also contribute to the signal variations at ± 1.6 ppm. In this study, we assessed the feasibility of using the asymmetry analysis method to quantify the NOE(‐1.6 ppm) by analyzing variations of these contamination effects at ± 1.6 ppm in an animal stroke model. If variations in these contaminations at ± 1.6 ppm in stroke are significantly smaller than the NOE(‐1.6 ppm) effect, it can be inferred that the asymmetry analysis method is feasible for quantifying the NOE(‐1.6 ppm).

## 2 MATERIALS AND METHODS

### 2.1 Asymmetry analysis

The MTR_asym_ metric for quantifying NOE is defined by,

(2) $${\text{MTR}}_{\text{asym}}\left(\Delta \omega \right)=\frac{S\left(-\Delta \omega \right)-S\left(+\Delta \omega \right)}{S_{0}}$$

where (+) represents the resonance frequency offset of the labeled protons; (‐) is the resonance frequency offset on the opposite side of the water peak; S is the measured CEST signal with RF saturation; $S_{0}$ is the control CEST signal acquired without RF saturation. Note that the direct subtraction of the S(−Δω) and S(+Δω) in Equation (2) cannot eliminate the DS and MT effects due to the shine‐through effect and R_1W_.^39^, ^40^ To address these limitations and further improve the specificity in quantifying CEST/NOE effects, an inverse subtraction analysis with correction of R_1W_, known as apparent exchange‐dependent relaxation (AREX), has been introduced for the extraction of CEST effects.^40^ Hence, we also combine AREX with the asymmetry analysis, termed AREX_asym_, to quantify the NOE effects,

(3) $${\text{AREX}}_{\mathrm{asym}}\left(\Delta \omega \right)=\left(\frac{S_{0}}{S\left(+\Delta \omega \right)}-\frac{S_{0}}{S\left(-\Delta \omega \right)}\right)R_{1w}\left(1+f_{m}\right)$$

where *f*_m_ is the MT pool concentration. Note that, for quantifying the APT effect with the MTR_asym_ and AREX_asym_ methods, the symbol (+) and (‐) in Equations (2) and (3) are replaced according to the resonance frequency offsets for the amide protons.

### 2.2 Lorentzian difference (LD) analysis

The LD analysis is an approach used to mitigate the DS and MT effects,^15^ which is similar to the role of the asymmetry analysis. LD can diminish both the symmetric and asymmetric MT effects, while the asymmetry analysis can only reduce the symmetric MT effect. Compared to the asymmetry analysis method, the LD analysis can provide separate estimations for the upfield and downfield CEST/NOE effects. This allows the evaluation of their relative contributions to the asymmetry analysis values. In this study, the label signal (*S*_L_) represents the measured CEST signal, and the reference signal for LD analysis (S_LD___R_) is estimated using a two‐pool (water and MT) model Lorentzian fitting of the CEST Z‐spectrum at specific frequency offsets of ± 4000, ± 3500, ± 3000, ± 2500, ± 150, ± 100, ± 50, and 0 Hz (‐13.3 to ‐8.3 ppm, ‐0.5 to 0.5 ppm, and 8.3 to 13.3 ppm at 7 T). Table 1 provides the starting points and boundaries of the LD analysis. The goodness of the LD fitting was assessed by the sum of squared errors. MTR_LD_ and AREX_LD_ spectra were obtained using Equations (2) and (3), respectively, with substitution of S_LD___R_(Δω) and S_L_(Δω) with S(‐Δω) and S(+Δω). To evaluate the variation of the CEST/NOE signals in stroke lesion, the MTR_LD_ difference spectrum (ΔMTR_LD_) and AREX_LD_ difference spectrum (ΔAREX_LD_) were obtained by subtracting two MTR_LD_ spectra and two AREX_LD_ spectra, respectively, between the contralateral normal tissue and stroke lesion. Comparing the ΔMTR_LD_ or ΔAREX_LD_ values at ± 1.6 ppm or the resonance frequency offsets of other pools could be informative in determining whether the variation in the NOE(‐1.6 ppm) effect dominates over the variation in other CEST/NOE effects. It is important to note that the asymmetry analysis method cannot eliminate the asymmetric MT effect. To assess the contribution of the variation in the asymmetric MT effect in stroke lesion, we first obtained the MTR_LD_asym_ and AREX_LD_asym_ spectra through the asymmetry analysis of MTR_LD_ and AREX_LD_ spectra, respectively. This process resulted in spectra free of contributions from the asymmetric MT effect, which we term LD asymmetry analysis spectra. We then compared the differences of MTR_asym_ and AREX_asym_ between stroke and normal tissue (ΔMTR_asym_, ΔAREX_asym_), which contain contributions from the asymmetric MT effect, with the differences of MTR_LD_asym_ and AREX_LD_asym_ between stroke and normal tissue (ΔMTR_LD_asym_, ΔAREX_LD_asym_). A relatively small difference between ΔMTR_LD_asym_ and ΔMTR_asym_, or between ΔAREX_LD_asym_ and ΔAREX_asym_, would indicate the negligible contribution from the variation in the asymmetric MT effect to the NOE(‐1.6 ppm) contrast using the asymmetry analysis method.

**TABLE 1:** The fitting parameters for the LD analysis and the multiple‐pool model Lorentzian fit methods.

	Start	Lower	Upper
A_water_	0.9	0.02	1
W_water_	1.4	0.3	10
Δ_water_	0	−1	1
A_amide_	0.01	0	0.3
W_amide_	0.5	0.4	3
Δ_amide_	3.5	3	4
A_amine_	0.01	0	0.3
W_amine_	1.5	0.5	5
Δ_amine_	2	1	3
A_NOE(‐1.6)_	0.01	0	0.3
W_NOE(‐1.6)_	1	0.5	1.5
Δ_NOE(‐1.6)_	−1.6	−2	−1
A_NOE(‐3.5)_	0.01	0	0.3
W_NOE(‐3.5)_	3	1	5
Δ_NOE(‐3.5)_	−3.5	−4.5	−2.5
A_MT_	0.1	0	1
W_MT_	25	10	100
Δ_MT_	0	−4	4

*Note*: Starting points and boundaries of the amplitude (A), the peak width (W), and the chemical shift (Δ) of each CEST/NOE/MT pool in the LD analysis and the multiple‐pool model Lorentzian fit. For the LD analysis, only water and MT pools were used. The unit of the peak width and the chemical shift is ppm.

### 2.3 Multiple‐pool Lorentzian fit

To further evaluate the specificity of the asymmetry analysis of the NOE(‐1.6 ppm) effect, we correlated it with the multiple‐pool Lorentzian fitted NOE(‐1.6 ppm) effect as well as other fitted CEST/NOE and MT effects. Equation (4) gives the model function of the multiple‐pool Lorentzian fit method.

(4) $$\frac{S\left(\Delta \omega \right)}{S_{0}}=1-\sum_{i=1}^{N}L_{i}\left(\Delta \omega \right)$$

Here, L_i_(Δω) = A_i_/(1+(Δω‐Δ_i_)^2^/(0.5W_i_)^2^), which represents a Lorentzian line with central frequency offset from water (Δ_i_), peak full‐width at half maximum (W_i_), and peak amplitude (A_i_). *N* is the number of fitted pools. A six‐pool model Lorentzian fit including amide, guanidine/amine, water, NOE(‐1.6 ppm), NOE(‐3.5 ppm), and MT was performed to process the Z‐spectra. Table 1 lists the starting points and boundaries of the fits based on our experiences and previous publications.^41^, ^42^ The label signal (S_mfit_L_) was obtained by the sum of all Lorentzians, and the reference signal (S_mfit_R_) for quantifying each CEST/NOE or MT effect was obtained by the sum of all Lorentzians except the corresponding CEST/NOE or MT pool.^43^ For the CEST/NOE effects, MTR_mfit_ and AREX_mfit_ spectra were obtained using Equations (2) and (3), respectively, with substitution of S_mfit___R_(Δω) and S_mfit_L_(Δω) with S(‐Δω) and S(+Δω). For the MT effect, MTR_mfit_ spectra were obtained using Equation (2), similar to that for the CEST/NOE effects, while AREX_mfit_ spectra were calculated using R_1w_·MTR_mfit_ /(1‐MTR_mfit_) (see derivation in the supporting information in Ref^44^). ΔMTR_mfit_ and ΔAREX_mfit_ spectra, representing the difference of MTR_mfit_ and AREX_mfit_ between stroke and normal tissue, were also obtained. The Lorentzian fitted guanidine/amine, NOE(‐1.6 ppm), NOE(‐3.5 ppm), and MT values were obtained with frequency offsets at 2 , ‐1.6 , ‐3.5 , and ‐2.3 ppm, respectively, in the corresponding fitted spectra. Table S1 lists the abbreviations and brief definitions of all the quantification metrics used in this study.

### 2.4 Animal preparation

Ischemic stroke was induced in the left hemispheres of the brains of five rats through middle cerebral artery occlusion (MCAO) as follows. First, the animals were anesthetized with 4% isoflurane (ISO) in an induction chamber and maintained at 2.0%–2.5% ISO during surgery. Then, a 30 mm, silicon‐coated, 4‐0 nylon suture (Doccol Corporation, Redlands, California, USA) was carefully inserted into the left internal carotid artery and advanced until it blocked the MCA at a depth of 18–20 mm. The suture was tightened around the filament to induce ischemic stroke, and the incision was closed. Immediately after surgery, the rats were transferred from the surgery room to the MRI scanner. During MRI scanning, the animals were anesthetized with a combination of 2% ISO and 98% O_2_. The breathing rate of the rats was continuously monitored throughout the experiments. The rectal temperature of the rats was maintained at approximately 37°C throughout the MRI experiments using a warm‐air feedback system. All experimental procedures were reviewed and approved by the Vanderbilt University Institutional Animal Care and Use Committee.

### 2.5 MRI

The experiments were conducted on a Varian DirectDrive horizontal 7T MRI system equipped with a 38 mm Doty RF coil (Doty Scientific Inc. Columbia, South Carolina, USA). The measurements involved the application of a continuous wave (CW) CEST preparation with an RF saturation length of 5 s and a saturation power (ω_1_) of 1 µT, followed by an imaging readout and a 2 s recovery time (TR = 7 s). Z‐spectra were acquired with frequency offsets at ± 4000, ± 3500, ± 3000, ± 2500, and from ‐1500 to 1500 Hz with a step size of 50 Hz (‐13.3 to 13.3 ppm on 7T). Control signals were obtained by setting the RF offset to 100 000 Hz (333 ppm on 7T). R_1W_ and f_m_ were obtained using a selective inversion recovery (SIR) quantitative MT method with TR of 2 s.^45^ The apparent diffusion coefficient (ADC) was obtained using the pulse gradient spin echo sequence with TR of 3 s. This involved applying gradients simultaneously on three axes, with a gradient duration of 6 ms, a separation of 12 ms, and five b‐values between 0 and 1000 s/mm^2^. All images were acquired using single‐shot spin‐echo echo planar imaging (SE‐EPI) with triple references for phase correction and with a TE of 28 ms, a matrix size of 64 × 64, a field of view (FOV) of 30 mm × 30 mm, a resolution of 0.47 mm × 0.47 mm, a slice thickness of 2 mm, and a receiver bandwidth of 250 kHz. The number of averages were 1, 4, and 1 for the CEST, quantitative MT, and ADC imaging, respectively. The scan times were around 16, 2, and 0.5 min for the CEST, quantitative MT, and ADC imaging, respectively. These images were acquired 1–3 days before (baseline) and at 0.5–1, 1–1.5, and 1.5–2 h after the MCAO surgery for the time‐course analysis.

### 2.6 Data analysis

All data analyses were conducted using MATLAB. For in vivo experiments, fitting methods were applied voxel by voxel, and the images were preprocessed by smoothing with a 3 × 3 median filter before the fitting process. The fitting was performed to achieve the lowest root mean square of residuals (RMSR) between the data and model. To assess the stroke lesion, regions of interest (ROIs) were manually delineated on the left hemispheres from the R_1w_ maps acquired at 1.5–2 h after the onset of stroke. These ROIs were then applied to all other parametric maps acquired at different time points for consistency in the analysis. For comparison, ROIs of the contralateral normal tissue were selected by mirroring the stroke ROIs. Student's *t*‐test was employed to evaluate differences in parameters, which were considered to be statistically significant when *p* < 0.05.

## 3 RESULTS

**FIGURE 1:**
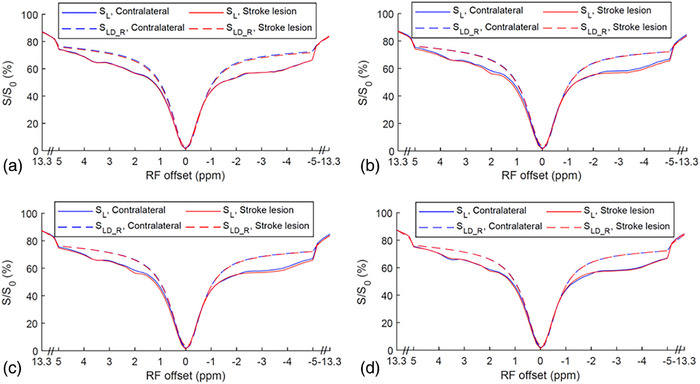
The average CEST Z‐spectra (S_L_) and the corresponding LD reference spectra (S_LD_R_) from stroke lesion (red) and contralateral normal tissues (blue) acquired before (a), 0.5–1 h (b), 1–1.5 h (c), and 1.5–2 h (d) after the onset of stroke.

**FIGURE 2:**
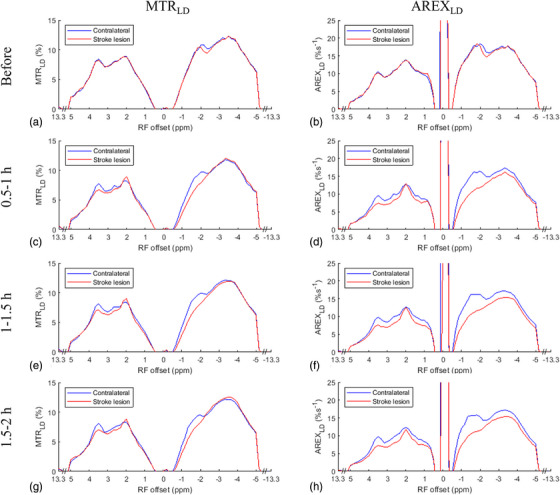
The average MTR_LD_ spectra (left column) and AREX_LD_ spectra (right column) from stroke lesion (red) and contralateral normal tissue (blue), respectively, acquired before (a, b), 0.5–1 h (c, d), 1–1.5 h (e, f), and 1.5–2 h (g, h) after the onset of stroke.

Figure 1 shows the average of CEST Z‐spectra and the corresponding LD reference spectra obtained from the stroke lesions and contralateral normal tissues of the five rats at various time points: before stroke induction and 0.5–1, 1–1.5 , and 1.5–2 h after the onset of stroke. Figure S1 displays the average of these CEST Z‐spectra, along with the standard deviation. The alignment of the CEST Z‐spectra and the LD reference spectra within ± 0.5 ppm and beyond ± 8.3 ppm indicate the successful fitting. The APT, guanidinium CEST, and NOE(‐3.5 ppm) effects are clearly observed on the CEST Z‐spectra, while the NOE(‐1.6 ppm) effect is not clearly distinguishable due to its overlap with the DS effect. Figure 2 illustrates the average of the fitted MTR_LD_ spectra and fitted AREX_LD_ spectra from the stroke lesions and the contralateral normal tissues, respectively. Figure S2 shows these spectra, along with the standard deviation. Notably, the NOE(‐1.6 ppm) peak becomes distinct and discernible in the LD fitted spectra. It is also observed that the NOE(‐1.6 ppm) peak is located at around ‐1.6 ppm in the AREX_LD_ spectra and it shifts to around ‐2 ppm in the MTR_LD_ spectra. The shift of NOE(‐1.6 ppm) peak is attributed to the influence of the significant DS effect in the MTR_LD_ spectra. In contrast, the DS effect in the AREX_LD_ spectra can be effectively removed, allowing for a more accurate characterization of the NOE(‐1.6 ppm) effect.

**FIGURE 3:**
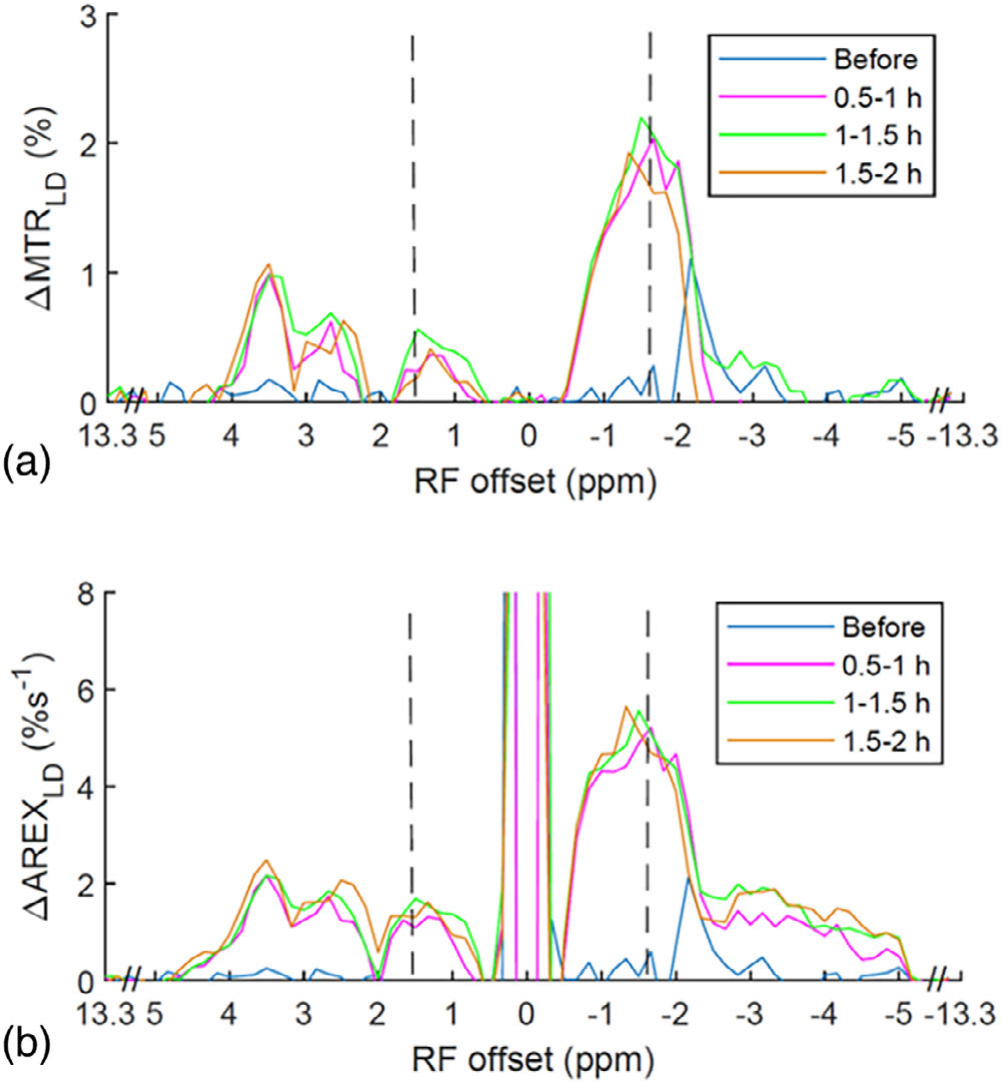
The average ΔMTR_LD_ spectra (a) and ΔAREX_LD_ spectra (b) acquired before, 0.5–1 , 1–1.5, and 1.5–2 h after the onset of stroke. Dashed lines show the RF frequency offsets at 1.6  and ‐1.6 ppm, respectively.

Figure 3 shows the average ΔMTR_LD_ spectra and ΔAREX_LD_ spectra. Figure S3 shows these spectra, along with the standard deviation. The ΔMTR_LD_ values at ‐1.6  and +1.6 ppm are shown as (2.0% vs. 0.3%), (2.1% vs. 0.3%), and (1.6% vs. 0.1%) for the different time points after the onset of stroke. Meanwhile, the ΔAREX_LD_ values at ‐1.6  and +1.6 ppm are (5.2%s^−1^ vs. 1.2%s^−1^), (5.1%s^−1^ vs. 1.4%s^−1^), and (4.7%s^−1^ vs. 1.3%s^−1^) at the corresponding time points. Notably, the ΔMTR_LD_ and ΔAREX_LD_ values at ‐1.6 ppm are significantly greater than those at +1.6 ppm after the onset of stroke. This suggests that the asymmetry analysis of NOE(‐1.6 ppm) in ischemic stroke primarily reflects the variation of NOE(‐1.6 ppm), rather than the guanidinium/amine CEST effect. The ΔMTR_LD_ and ΔAREX_LD_ spectra show a dip at 2 ppm, which could be explained by the cancellation effect from both the guanidinium CEST effect and the amine CEST effect. By comparing the ΔMTR_LD_ and ΔAREX_LD_ values at ‐1.6 ppm with those near 2 ppm (e.g., 3 , 1 ppm) where the broad amine CEST effect is present but the guanidinium CEST effect is small, it can be further concluded that the variation of NOE(‐1.6 ppm) dominates over either the guanidinium CEST or the amine CEST effect. The ΔMTR_LD_ and ΔAREX_LD_ values at 3 ppm are (0.3% and 1.2%s^−1^), (0.5% and 1.5%s^−1^), and (0.5% and 1.6%s^−1^) for the different time points after the onset of stroke. The ΔMTR_LD_ and ΔAREX_LD_ values at 1 ppm are (0.2% and 0.8%s^−1^), (0.4% and 1.4%s^−1^), and (0.2% and 0.9%s^−1^) for the different time points after the onset of stroke. These values are considerably lower than the corresponding ΔMTR_LD_ and ΔAREX_LD_ values at ‐1.6 ppm. The ΔMTR_LD_ and ΔAREX_LD_ values at ‐3.5 ppm are (‐0.1% and 1.2%s^−1^), (0.1% and 1.6%s^−1^), and (‐0.4% and 1.5%s^−1^) for the different time points after the onset of stroke. These values are also considerably smaller than the ΔMTR_LD_ and ΔAREX_LD_ values at ‐1.6 ppm, suggesting that the NOE(‐3.5) effect has a negligible contribution to the CEST/NOE variation at ‐1.6 ppm in stroke can be negligible.

**FIGURE 4:**
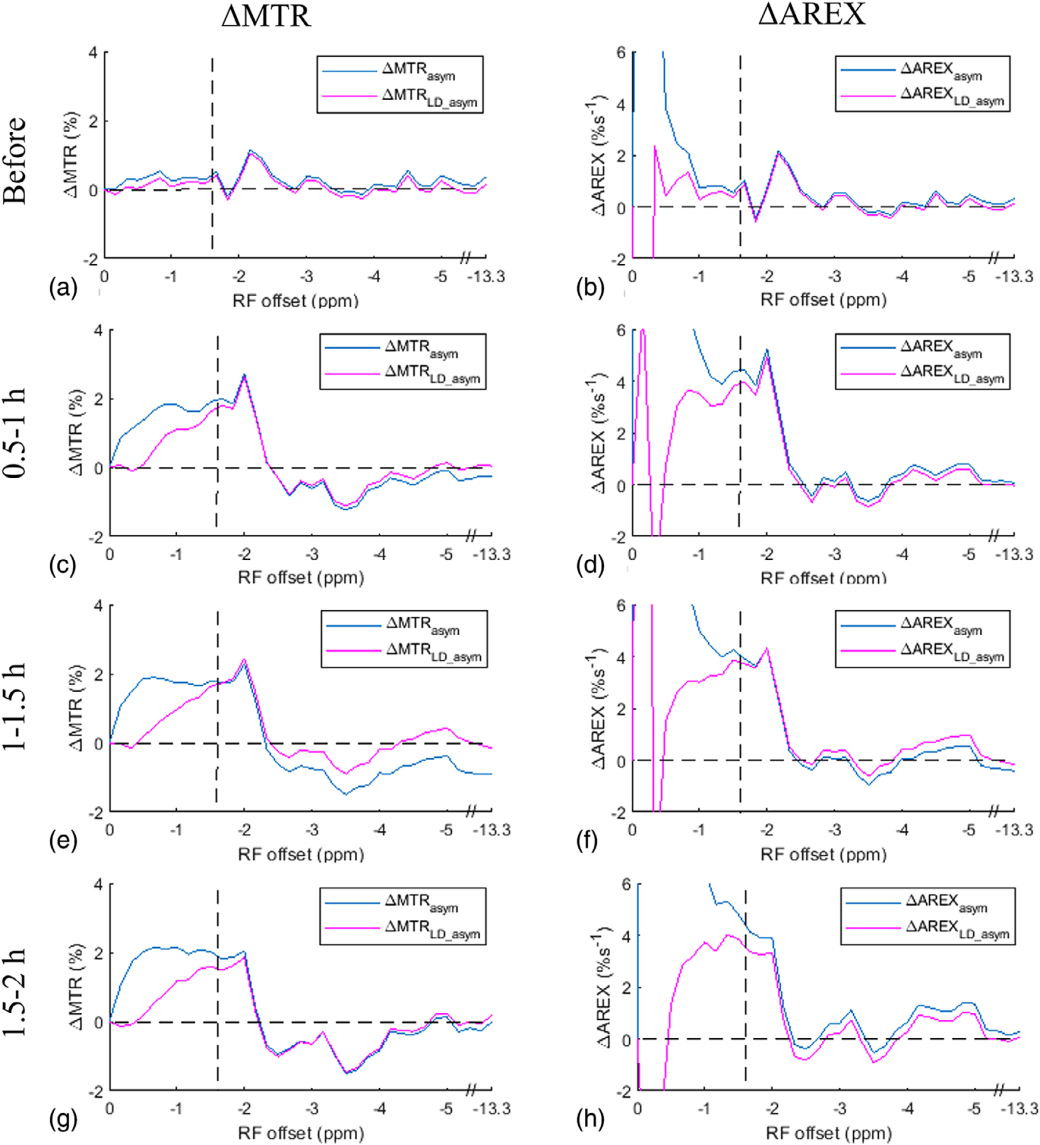
Comparison between the average ΔMTR_asym_ and ΔMTR_LD_asym_ spectra (left column), as well as between the average ΔAREX_asym_ and ΔAREX_LD_asym_ spectra (right column), respectively, acquired before (a, b), 0.5–1 h (c, d), 1–1.5 h (e, f), and 1.5–2 h (g, h) after the onset of stroke. Dashed lines represent the ΔMTR values of 0% or ΔAREX values of 0%s^−1^ and RF frequency offsets at ‐1.6 ppm.

Figure 4 compares the average ΔMTR_LD_asym_ and ΔAREX_LD_asym_ spectra with the average ΔMTR_asym_ and ΔAREX_asym_ spectra. Figure S4 further displays these spectra, along with the standard deviation. Figure S5 shows the corresponding average MTR_LD_asym_ and AREX_LD_asym_ spectra, as well as MTR_asym_ and AREX_asym_ spectra, from the stroke lesions and the contralateral normal tissues. Notably, these spectra in Figure 4 exhibit a close match at around ‐1.6 ppm, indicating that the variation of the asymmetric MT effect in the stroke lesion has a minor contribution to the asymmetry analysis of NOE(‐1.6 ppm). Both Figures 3 and 4 provide evidence supporting that $\Delta MTR_{asym}^{\prime }$ for quantifying NOE(‐1.6 ppm) can be ignored, and so, the asymmetry analysis method can be exploited to detect the NOE(‐1.6 ppm) effects in ischemic stroke.

**FIGURE 5:**
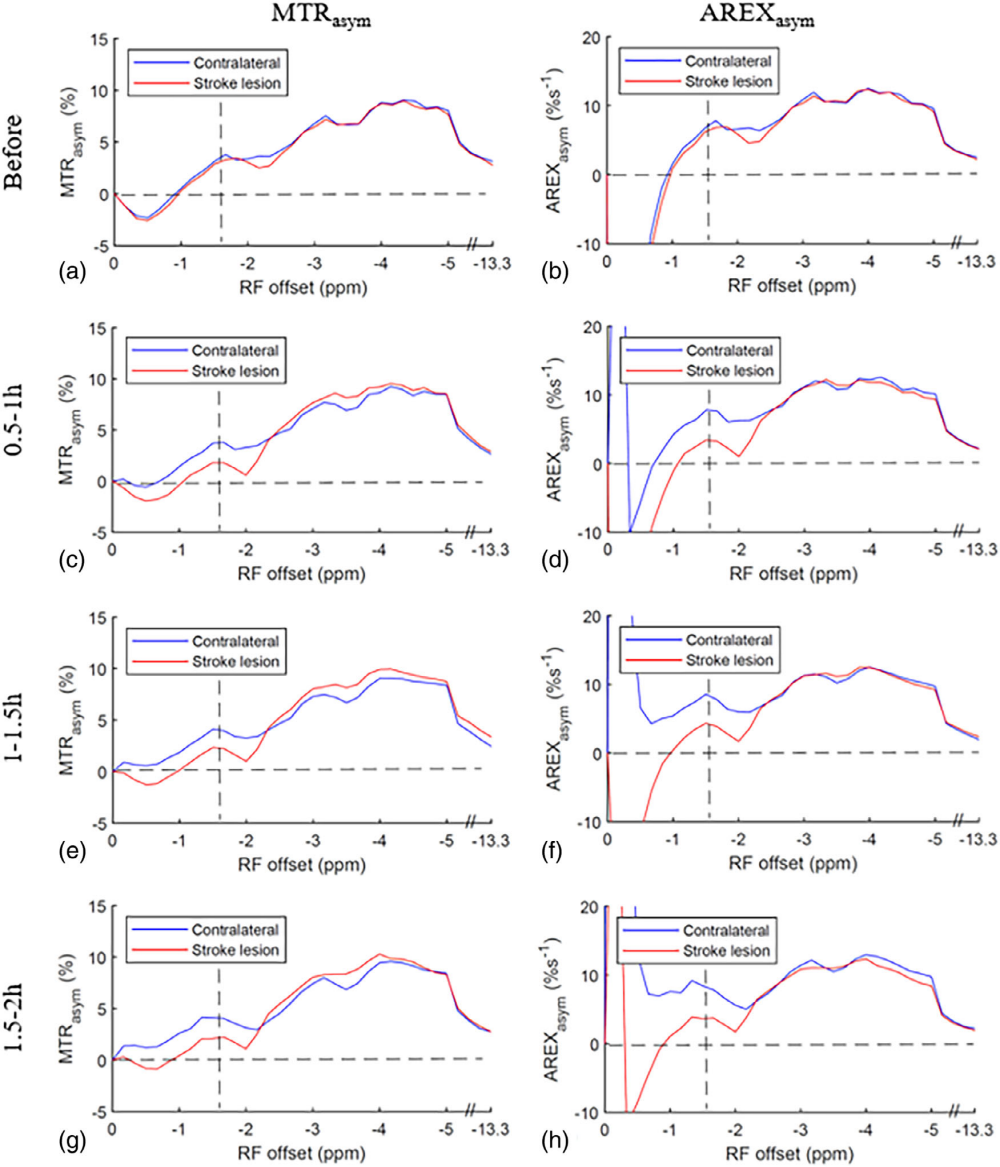
Average MTR_asym_ spectra (left column) and AREX_asym_ spectra (right column) from stroke lesion (red) and contralateral normal tissue (blue), respectively, acquired before (a, b), 0.5–1 h (c, d), 1–1.5 h (e, f), and 1.5–2 h (g, h) after the onset of stroke. Dashed lines represent the MTR_asym_ values of 0% or AREX_asym_ values of 0%s^−1^ and RF frequency offsets at ‐1.6 ppm.

Figure 5 shows the average MTR_asym_ spectra and AREX_asym_ spectra obtained from the stroke lesions and the contralateral normal tissues, respectively. Figure S6 shows these spectra, along with the standard deviation. Notably, the peaks observed at around ‐1.6 ppm should mainly originate from the NOE(‐1.6 ppm) effect. Additionally, a broad peak centered at around ‐3.5 ppm is observed, which is attributed to the NOE(‐3.5 ppm) effect. Furthermore, small dips are seen at ‐3.5 ppm, superimposed on the broad peak, which reflects the APT effect.

**FIGURE 6:**
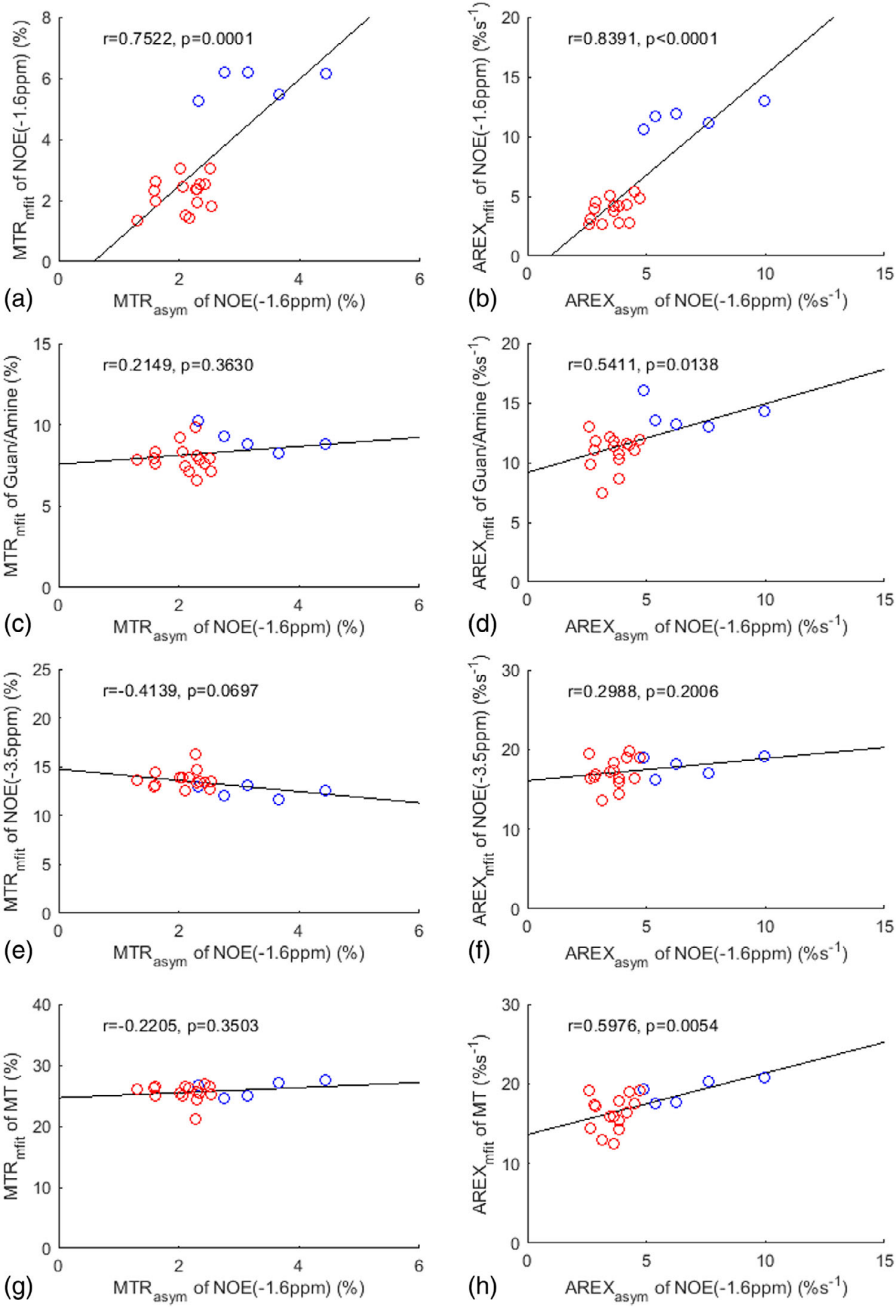
Summarized correlations of MTR_asym_ and AREX_asym_ quantified NOE(‐1.6 ppm) with MTR_mfit_ and AREX_mfit_ quantified NOE(‐1.6 ppm) (a, b), guanidine/amine CEST (c, d), NOE(‐3.5 ppm) (e, f), and MT (g, h) from stroke lesions acquired both before (blue circles) and at different time points after (red circles) the stroke onset. The Spearman's rank correlation coefficient (*r*) and *p* value of each correlation are provided. The black lines represent the linear regression of all data points in each correlation subfigure.

**FIGURE 7:**
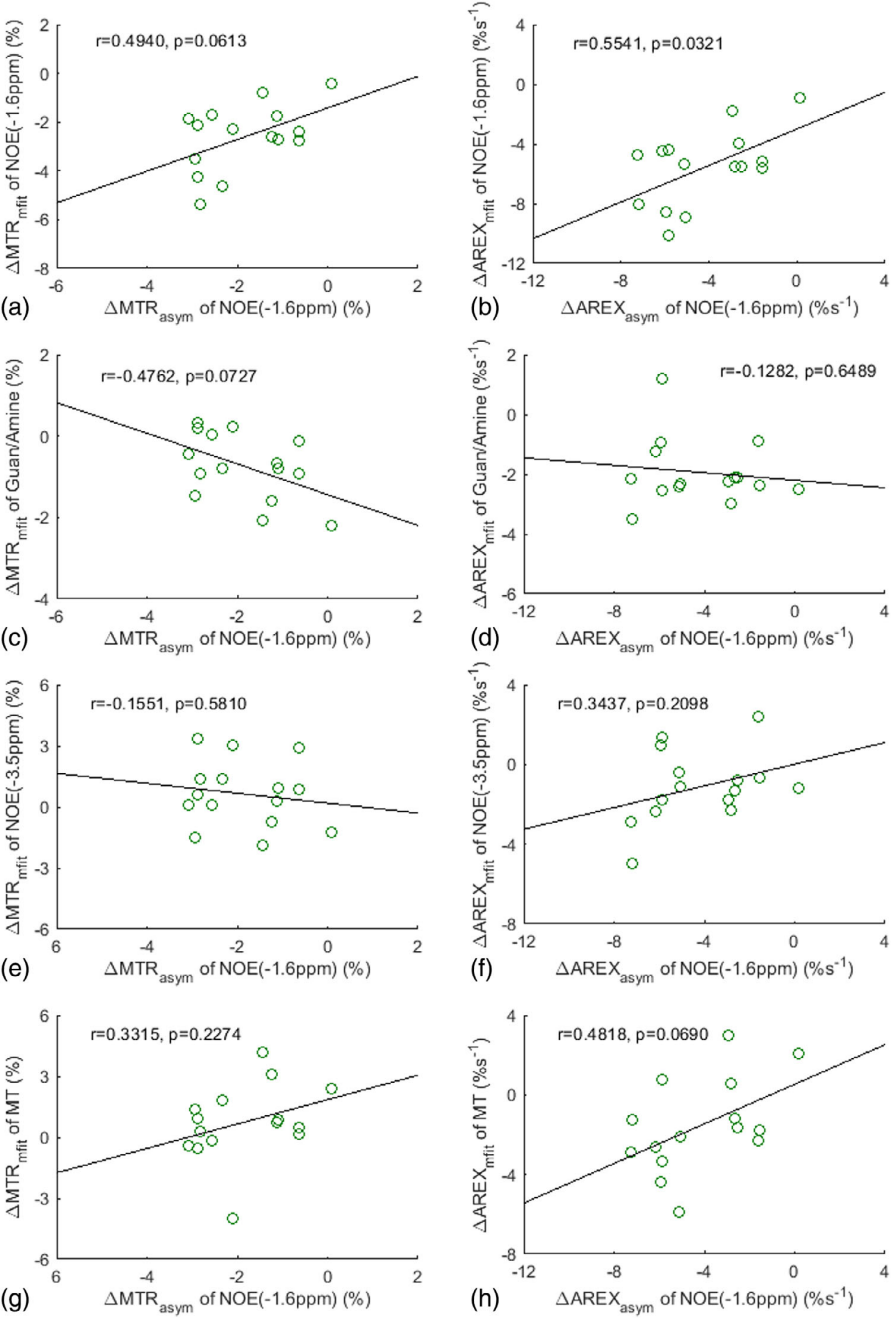
Summarized correlation of ΔMTR_asym_ and ΔAREX_asym_ quantified NOE(‐1.6 ppm) with ΔMTR_mfit_ and ΔAREX_mfit_ quantified NOE(‐1.6 ppm) (a, b), guanidine/amine CEST (c, d), NOE(‐3.5 ppm) (e, f), and MT (g, h) from stroke lesions acquired at different time points after the stroke onset (green circles). The Spearman's rank correlation coefficient (*r*) and *p* value of each correlation are provided. The black lines represent the linear regression of all data points in each correlation subfigure.

Figure 6 shows the correlation between the asymmetry analysis of the NOE(‐1.6 ppm) effect and the multiple‐pool Lorentzian fitted NOE(‐1.6 ppm), guanidine/amine CEST, NOE(‐3.5 ppm), and MT effects from stroke lesions, acquired both before and at various time points after the stroke onset. Tables S2‐S11 list these values derived from the asymmetry analysis and the multiple‐pool Lorentzian fit. It is noted that there is a significant correlation between the MTR_asym_ quantified NOE(‐1.6 ppm) effect and MTR_mfit_ quantified NOE(‐1.6 ppm), but not with MTR_mfit_ quantified guanidine/amine, NOE(‐3.5 ppm), and MT effects. Additionally, AREX_asym_ quantified NOE(‐1.6 ppm) also has a significant correlation with AREX_mfit_ quantified NOE(‐1.6 ppm) effect, but not with NOE(‐3.5 ppm) effect. AREX_asym_ quantified NOE(‐1.6 ppm) has a significant correlation with AREX_mfit_ quantified guanidine/amine CEST effect (*p* = 0.0138) and MT effect (*p* = 0.0054), but with a much higher *p*‐value than the correlation with AREX_mfit_ quantified NOE(‐1.6 ppm) (*p* < 0.0001). Figure 7 shows the correlation between ΔMTR_asym_ and ΔAREX_asym_ quantified NOE(‐1.6 ppm) effect and ΔMTR_mfit_ and ΔAREX_mfit_ quantified NOE(‐1.6 ppm), guanidine/amine CEST, NOE(‐3.5 ppm), and MT effects from stroke lesions acquired at various time points after the stroke onset. Notably, ΔAREX_asym_ quantified NOE(‐1.6 ppm) effect significantly correlates with ΔAREX_mfit_ quantified NOE(‐1.6 ppm) effect, but not with ΔAREX_mfit_ quantified guanidine/amine CEST, NOE(‐3.5 ppm), and MT effects. ΔMTR_asym_ quantified NOE(‐1.6 ppm) effect does not have a significant correlation with ΔMTR_mfit_ quantified NOE(‐1.6 ppm) effect, guanidine/amine CEST, NOE(‐3.5 ppm), and MT effects. However, it does correlate with ΔMTR_mfit_ quantified NOE(‐1.6 ppm) with a small *p*‐value (0.0613). These findings further suggest that asymmetry analysis at ‐1.6 ppm in stroke lesions primarily reflects the variation in the NOE(‐1.6 ppm) effect, rather than the contaminations from the guanidine/amine CEST, NOE(‐3.5 ppm), and MT effects.

**FIGURE 8:**
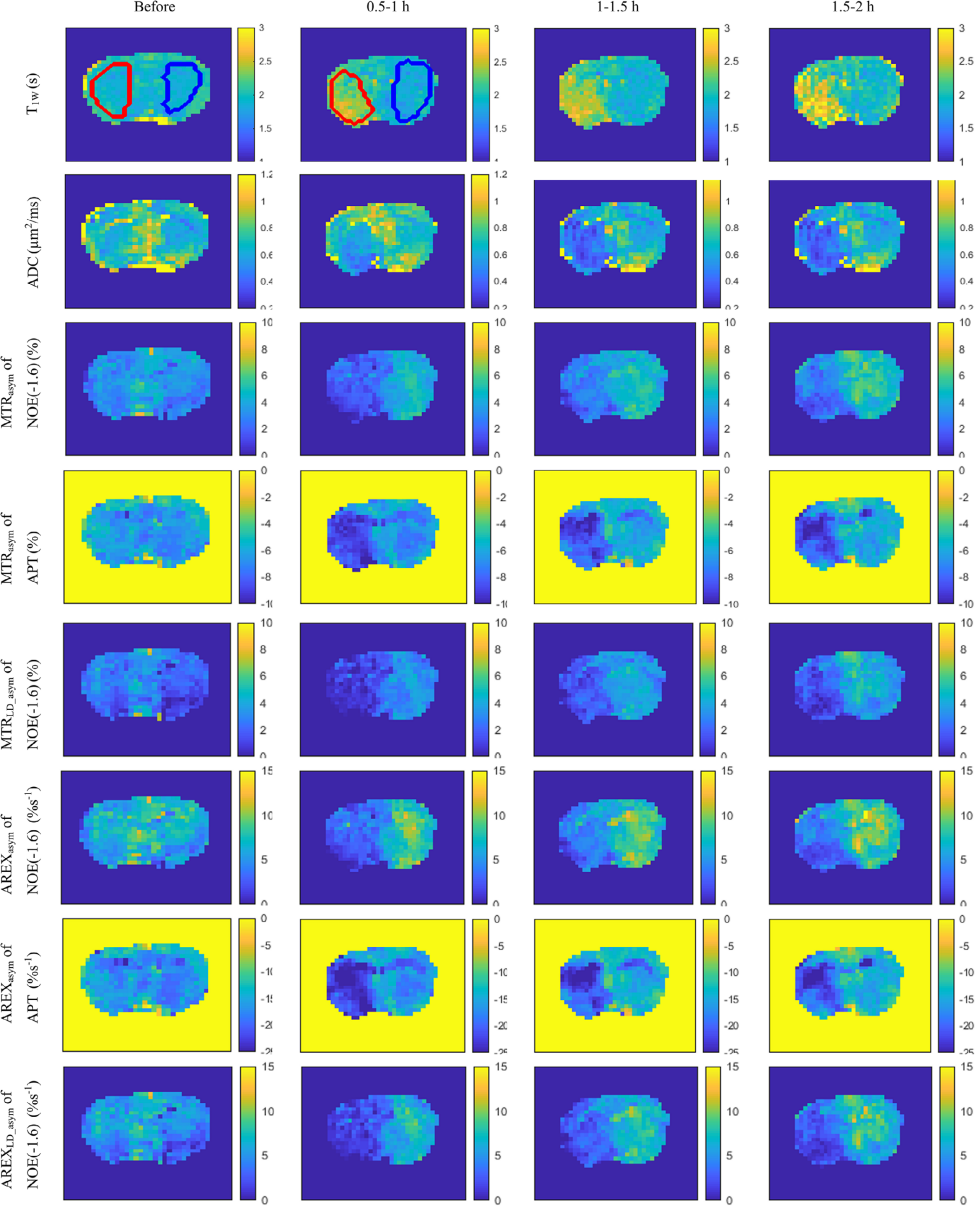
Maps of T_1w_, ADC, MTR_asym_ quantified NOE(‐1.6 ppm) and APT, MTR_LD_asym_ quantified NOE(‐1.6 ppm), AREX_asym_ quantified NOE(‐1.6 ppm) and APT, as well as AREX_LD_asym_ quantified NOE(‐1.6 ppm) acquired before and at different time points after ischemic stroke from the brain of a representative rat. ROIs of lesions and contralateral normal tissue are shown in T_1w_ maps.

**FIGURE 9:**
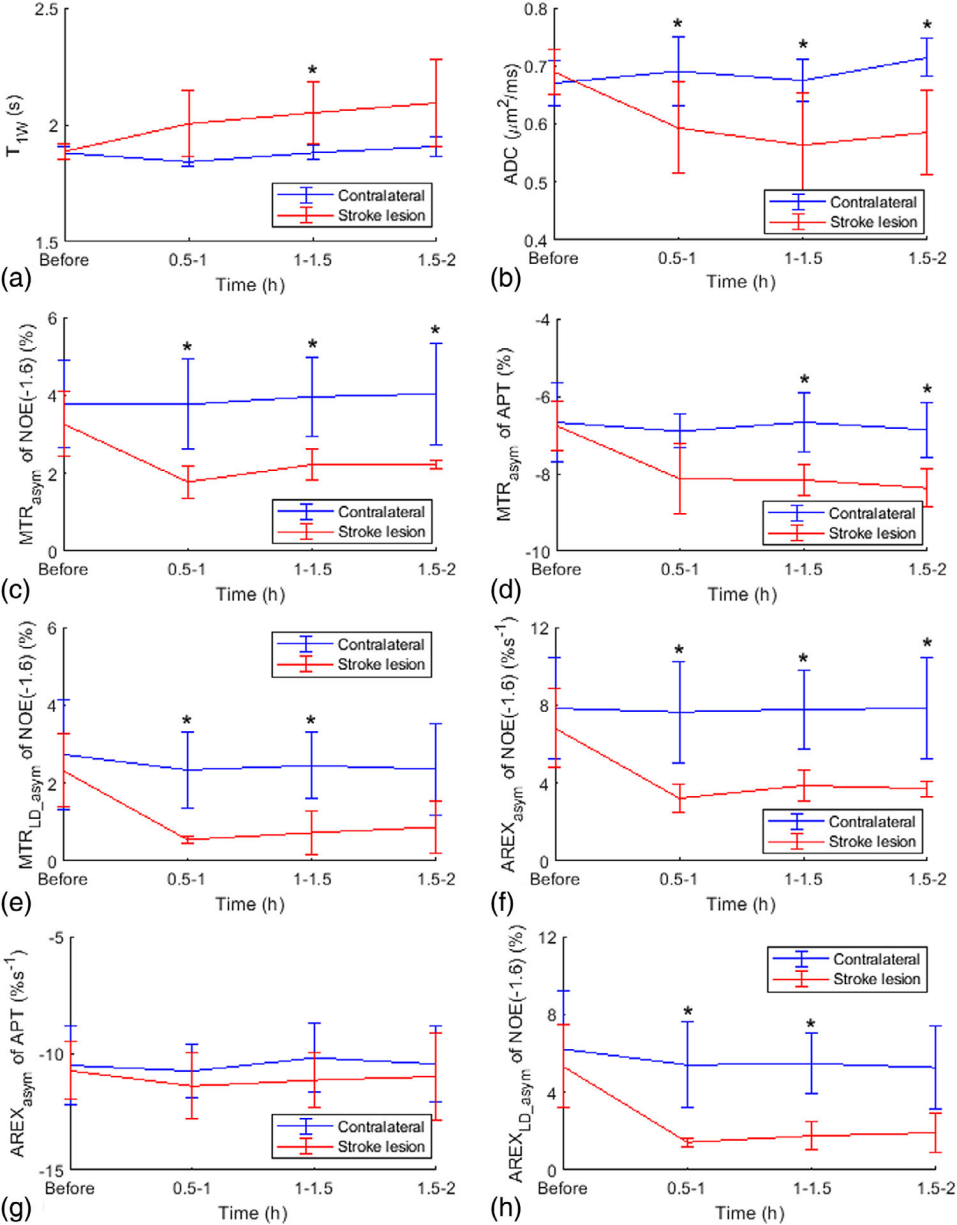
Time‐dependent statistics of T_1w_ (a) ADC (b), MTR_asym_ quantified NOE(‐1.6) (c), MTR_asym_ quantified APT (d), MTR_LD_asym_ quantified NOE(‐1.6 ppm) (e), AREX_asym_ quantified NOE(‐1.6 ppm) (f), AREX_asym_ quantified APT (g), and AREX_LD_asym_ quantified NOE(‐1.6 ppm) in stroke lesions (red) and contralateral normal tissues (blue) of five rats. (^*^ *p* < 0.05).

Figure 8 displays maps of T_1w_, ADC, MTR_asym_ quantified NOE(‐1.6 ppm) and APT, MTR_LD_asym_ quantified NOE(‐1.6 ppm), AREX_asym_ quantified NOE(‐1.6 ppm) and APT, as well as AREX_LD_asym_ quantified NOE(‐1.6 ppm) from the brain of a representative rat after the onset of stroke. Figure S7 shows maps of MTR_mfit_, MTR_LD_, AREX_mfit_, and AREX_LD_ quantified NOE(‐1.6 ppm) from this rat. The stroke lesion is visible in the left hemispheres on the ADC map after the onset of stroke. The lesion contrast in the NOE(‐1.6 ppm) map using the asymmetry analysis is similar to that using the LD asymmetry analysis and the multiple‐pool Lorentzian fit, but is different from the APT map using the asymmetry analysis. Figures 9 and S8 exhibit the time‐dependent statistics of these parameters or metrics in both the stroke lesions and contralateral normal tissues of five rats. Tables S12–S17 list the corresponding values. The ADC shows significant decreases at 0.5–1 h (*p* = 0.007), 1–1.5 h (*p* = 0.015), and 1.5–2 h (*p* = 0.012) after the onset of stroke. Conversely, T_1w_ shows a significant increase only 1–1.5 h (*p* = 0.046) after the onset of stroke. It is also observed that both MTR_asym_ and AREX_asym_ quantified NOE(‐1.6 ppm) show significant decreases at 0.5–1 h (*p* = 0.023, 0.020), 1–1.5 h (*p* = 0.004, 0.005), and 1.5–2 h (*p* = 0.037, 0.035) after the onset of stroke. In contrast, the MTR_asym_ quantified APT demonstrates significant decreases only 1–1.5 h (*p* = 0.022) and 1.5–2 h (*p* = 0.022) after the onset of stroke. The AREX_asym_ quantified APT, however, shows no significant changes after the onset of stroke. Moreover, NOE(‐1.6 ppm) values using the asymmetry analysis show roughly similar time dependence as those using the LD asymmetry analysis and the multiple‐pool Lorentzian fit.

## 4 DISCUSSION

The asymmetry analysis method has been widely used in CEST imaging with an assumption that the variations of the upfield NOE effects in pathologies can be ignored.^6^, ^9^, ^38^ However, it is difficult to assess the variations of individual CEST/NOE effects by directly observing the CEST Z‐spectrum due to the fluctuations of the confounding baseline signals induced by the variations of T_1W_, DS, and MT effects in specific pathologies. The ΔAREX_LD_ spectra can effectively reduce the contributions from T_1W_ and MT effects, allowing a relatively specific estimate of the variations in individual CEST/NOE effects. However, few studies have validated the feasibility of asymmetry analysis previously. For the asymmetry analysis of the CEST/NOE effect at ‐1.6 ppm in our study, it may involve contributions from the NOE(‐1.6 ppm), NOE(‐3.5 ppm), guanidinium/amine CEST, and asymmetric MT effect. Through the LD analysis, we have successfully verified that the variation of NOE(‐1.6 ppm) effect has a dominant contribution at ‐1.6 ppm compared with other effects in stroke lesions present in the asymmetry analysis spectrum. This finding provides evidence supporting that the asymmetry analysis method is capable of characterizing the changes in the NOE(‐1.6 ppm) effect in ischemic stroke.

In this study, our proposed method is conducted on an animal MRI scanner with small B_0_ shifts, the asymmetry analysis method utilized signals acquired at two frequency offsets at ± 3.5 ppm and a control scan (0.7 min). For a human MRI scanner with relatively larger B_0_ shifts, the asymmetry analysis method can be performed with a six‐offset acquisition scheme for B_0_ correction: that is, CEST signals at ± 3 , ± 3.5 , and ± 4 ppm, along with a control signal (1.6 min).^46^ In contrast, the multiple‐pool Lorentzian fit method necessitates the acquisition of the entire Z‐spectrum, which involves a CEST image acquisition with 69 frequency offsets and a control scan (16 min). Additionally, the LD method requires a CEST acquisition of 15 frequency offsets for fitting the background signals in this study, at least three frequency offsets near 3.5 ppm for APT imaging and B_0_ correction, and a control scan (4.4 min). Thus, the asymmetry analysis with B_0_ correction would take around 10% and 37% of the time needed for the multiple‐pool Lorentzian fit and the LD analysis, respectively. Therefore, the asymmetry analysis could significantly reduce total imaging time. Additionally, it seems that the use of a few CEST signals in the asymmetry analysis would result in lower SNR images compared to the multiple‐pool Lorentzian fit. However, results in Figures 8 and S7 show that the asymmetry analysis does not exhibit dramatically reduced SNR compared to the multiple‐pool Lorentzian fit. This is because most of the CEST signals used in the multiple‐pool Lorentzian fit are not at around ‐1.6 ppm and thus do not contain information about the underlying NOE(‐1.6 ppm) effect. Moreover, the fitting approaches are not robust when SNR is low. This issue becomes more severe in human imaging at a 3T scanner, where most CEST/NOE and DS effects significantly overlap. However, without using fitting approaches, the asymmetry analysis can enhance the robustness of quantifying the NOE(‐1.6 ppm), making it more suitable for the NOE(‐1.6 ppm) imaging at a relativity lower magnetic field. Consequently, this could be beneficial to clinical applications and further studies on the contrast mechanism of NOE(‐1.6 ppm) in ischemic stroke.

The LD analysis used in our study may underestimate both the CEST and NOE effects due to the use of CEST signals within ± 0.5 ppm as reference. However, this potential quantification bias should not significantly influence our conclusion because the subtraction of the LD spectra between stroke lesions and contralateral normal tissues could reduce the bias. The fitting methods used in the LD analysis and the multiple‐pool Lorentzian fit are not robust when the SNR of the CEST signals is low. However, for validation purposes, this issue can be addressed by choosing a large ROI from the lesions and the normal tissues. Additionally, our studies were conducted at 7T, in which the DS effect at ± 1.6 ppm is relatively weak. At lower magnetic fields, the DS effects would be more pronounced, and as a result, the NOE(‐1.6 ppm) signal may be diluted. However, the CEST effect at +1.6 ppm is also expected to experience a similar dilution due to the influence of the DS effect. Consequently, the relative contribution from guanidinium/amine CEST and the NOE(‐1.6 ppm) effect is not expected to vary significantly at lower fields. In our experiments, we used an RF saturation power of 1µT. With lower saturation powers, the NOE effect would also be greater than the guanidinium/amine CEST effect, considering that the NOE coupling rate is much slower than the guanidine/amine‐water exchange rate.^47^ On the other hand, at higher saturation powers, the guanidinium/amine CEST is expected to become more prominent. Therefore, we suggest using relatively lower saturation powers for the asymmetry analysis of NOE(‐1.6 ppm) imaging. The difference between ΔMTR_LD_asym_ and ΔMTR_asym_, as well as between ΔAREX_LD_asym_ and ΔAREX_asym_, at around and below ‐1 ppm in Figure 4 may be due to B_0_ shift or other unknown effects that are insensitive to the LD analysis since it uses CEST signal within ± 0.5 ppm as reference signals. However, such differences can be effectively characterized using the asymmetry analysis method.

During ischemic stroke, the inadequate blood supply leads to anaerobic glycolysis and tissue acidosis, initiating a cascade of biochemical events that eventually result in membrane breakdown and neuronal death, known as the ischemic cascade.^48^ Imaging these biochemical events may lead to an accurate diagnosis of stroke and enable the monitoring of therapeutic interventions. The APT arises from mobile proteins/peptides,^6^ and the guanidinium CEST arises from the protein arginine and creatine.^7^, ^8^ These two CEST effects are in the slow and intermediate exchange regimes, respectively, making them highly sensitive to tissue pH variation. Consequently, APT^6^, ^18^, ^19^, ^20^, ^21^ and guanidinium CEST^22^, ^30^ imaging have been previously employed to enhance the detection of viable ischemic penumbra. The NOE(‐1.6 ppm) and NOE(‐3.5 ppm) arise from different sources, with NOE(‐1.6 ppm) originating from membrane choline phospholipids,^11^, ^12^ and NOE(‐3.5 ppm) arising from mobile macromolecular components with finite linewidth, including large proteins and membrane phospholipids.^13^, ^14^ Recent studies have indicated that NOE(‐1.6 ppm) can be enhanced when rats breathe pure oxygen instead of normal air,^49^ which may be also related to the blood supply.^50^, ^51^ Another recent study has also reported the decreased NOE(‐1.6 ppm) signals in ischemic stroke, and interpreted it as an indication of lipid peroxidation and membrane damage.^52^ The precise mechanism underlying the variations of the NOE(‐1.6 ppm) in stroke is not fully understood, but it may be explained by changes in the states of the cell membrane or blood oxygenation according to previous studies.^49^, ^50^ Further investigations are required to elucidate the mechanism of NOE(‐1.6 ppm) in stroke lesions, and the development of a rapid and robust NOE(‐1.6 ppm) quantification method is expected to facilitate such studies.

In this study, rats inhaled oxygen during a 2 h MRI scan. It is essential to note that the significant NOE(‐1.6 ppm) changes in this study are linked to the breathing of oxygen. Our previous studies indicate that the NOE(‐1.6 ppm) signal in normal tissues can increase by 20% when breathing oxygen as opposed to air.^49^, ^51^ In experiments with breathing air, the NOE(‐1.6 ppm) contrast between normal and ischemic tissue would likely be smaller. While prolonged oxygen inhalation can be toxic, oxygen therapy has proven to increase oxygen supply to ischemic tissues. This effectively reduces both infarct volume and neurological deficits, and it also improves outcomes after cerebral ischemia/reperfusion.^53^

Since MT effects in the acute stage should have no significant changes (the mean *f*_m_ values from stroke lesion and contralateral normal tissues are 8.9 ± 0.3% vs. 8.7 ± 0.3%, 8.8 ± 0.3% vs. 8.6 ± 0.3%, 9.0 ± 0.5% vs. 9.0 ± 0.5%, and 8.4 ± 0.8% vs. 8.6 ± 0.8% at different time points after the stroke onset), the main factor that contributes the difference between MTR_asym_ and AREX_asym_ should be T_1W_. By comparing the MTR_asym_ and AREX_asym_ quantified NOE(‐1.6 ppm) in Figure 9, we observed similar patterns. Thus, this result suggests that the contribution from the variations in T_1W_ to the MTR_asym_ quantified NOE(‐1.6 ppm) contrasts should be negligible in stroke lesions. Figure S9 shows no significant correlations between T_1W_ and MTR_asym_ quantified NOE(‐1.6 ppm) as well as between T_1W_ and AREX_asym_ quantified NOE(‐1.6 ppm), which supports this finding. Considering that the AREX method requires an additional acquisition of T_1W_, we suggest utilizing the MTR_asym_ method to quantify the NOE(‐1.6 ppm) in the acute stage of ischemic stroke when the variations in T_1W_ and MT effect are relatively not significant.

In Figure 3, it is evident that the ΔMTR_LD_ at 3.5 ppm dominates over the ΔMTR_LD_ at ‐3.5 ppm. In contrast, the ΔAREX_LD_ at 3.5 ppm is comparable to that at ‐3.5 ppm. As a result, in Figure 9d and 9f, the MTR_asym_ quantified APT effect exhibits significant variations in stroke lesions, whereas the AREX_asym_ quantified APT effect shows no significant variations. This difference may be attributed to the ability of AREX metric to provide a more specific estimation of CEST/NOE effects without contaminations from T_1W_. Consequently, the insignificant variations observed in the AREX_asym_ quantified APT effect could be explained by the cancellation of variations in APT and NOE(‐3.5 ppm) in the stroke lesions. Figure S10 shows the MTR_asym_ and AREX_asym_ spectra, demonstrating the overlapping of APT on the NOE(‐3.5 ppm) peaks. On the other hand, the MTR_asym_ quantified APT may be significantly influenced by contributions from T_1W_. Previously, the AREX metric, in conjunction with a three‐point method that is more specific to APT than the asymmetry analysis, has been utilized to quantify APT in ischemic stroke, demonstrating a hypointense signal in the stroke lesion.^34^, ^54^ We conducted a similar analysis, and the results are presented in Figure S11. The findings align with those of previous publications, validating the consistency of our data with earlier reports.

In Figure S10, MTR_asym_ demonstrates a larger contrast between normal and ischemic tissue at around 1.6 ppm than that at 3.5 ppm. A previous study at 9.4 T also observed noticeable MTR_asym_ contrast between normal and ischemic tissue at around 1.6 ppm.^30^ However, in other previous studies conducted at 4.7 T, MTR_asym_ demonstrated a larger contrast at 3.5 ppm than at 1.6 ppm.^26^, ^55^ These different results may be attributed to the different magnetic fields used. It could be also due to the use of oxygen in our study, which may reduce the APT contrast and enhance the NOE(‐1.6 ppm) contrast. Additionally, the choice of ROI could be another reason for the differences observed. Further investigation into these differences is warranted.

## 5 CONCLUSION

In this study, we demonstrate the feasibility of using the asymmetry analysis method for quantifying the NOE(‐1.6 ppm) effect in ischemic stroke. The method effectively reflects the variations in NOE(‐1.6 ppm) while minimizing contributions from other effects. Compared with the multiple‐pool Lorentzian fit approaches, the asymmetry analysis would be a rapid and robust method to quantify the variations of NOE(‐1.6 ppm) in ischemic stroke.

## CONFLICT OF INTEREST STATEMENT

The authors declare no conflicts of interest.

## Supporting information



Supporting Information
