# Supplementary material for: Asymmetry analysis of nuclear Overhauser enhancement effect at ‐1.6 ppm in ischemic stroke
Source: Med Phys. 2025 Feb 11;52(5):2922–37. doi: 10.1002/mp.17677 (PMC12059535; doi:10.1002/mp.17677)
Supplement: Supplementary file 1 — Supporting Information [file MP-52-2922-s001.pdf]

Supporting information

Table S1 List of the abbreviations and brief definitions of all the quantification metrics used in this paper.

Abbreviation	Brief definition
MTR_{asym}	magnetization transfer ratio derived from an asymmetry analysis
$AREX_{asym}$	apparent exchange-dependent relaxation derived from an asymmetry analysis
MTR_{LD}	magnetization transfer ratio derived from Lorentzian difference analysis
$AREX_{LD}$	apparent exchange-dependent relaxation derived from Lorentzian difference analysis
MTR_{LD_asym}	the asymmetry analysis of MTR_{LD}
$AREX_{LD\_asym}$	the asymmetry analysis of $AREX_{LD}$ spectra
MTR_{mfit}	magnetization transfer ratio derived from a multiple-pool Lorentzian fit
$AREX_{mfit}$	apparent exchange-dependent relaxation derived from a multiple-pool Lorentzian fit
ΔMTR	difference spectrum of MTR
ΔAREX	difference spectrum of AREX

Table S2 List of the MTR_{asym} quantified NOE(-1.6ppm) values from contralateral normal tissues (N) and stroke lesions (L) of five rat brains.

rat	Before		0.5-1h		1-1.5h		1.5-2h	
	N (%)	L (%)	N (%)	L (%)	N (%)	L (%)	N (%)	L (%)
#1	4.7613	3.6691	5.0272	2.0959	5.0949	2.5237	5.1566	2.0673
#2	5.1246	4.4444	4.1810	1.2993	3.8509	2.4179	2.0904	2.1630
#3	3.2077	3.1357	4.4774	1.6034	4.8717	2.5337	5.1229	2.2901
#4	3.2897	2.7600	2.9457	2.3027	2.8406	1.6042	3.4179	2.3342
#5	2.5012	2.3220	2.2097	1.5860	3.1543	2.0256	4.3882	2.2820

Table S3 List of the $AREX_{asym}$ quantified NOE(-1.6ppm) values from contralateral normal tissues (N) and stroke lesions (L) of five rat brains.

rat	before		0.5-1h		1-1.5h		1.5-2h	
	N ($\%s^{-1}$)	L ($\%s^{-1}$)	N ($\%s^{-1}$)	L ($\%s^{-1}$)	N ($\%s^{-1}$)	L ($\%s^{-1}$)	N ($\%s^{-1}$)	L ($\%s^{-1}$)
#1	9.9654	7.6007	11.0238	3.8408	10.6338	4.5036	10.8846	3.6425
#2	11.1506	9.9410	8.4752	2.6178	7.6502	4.7038	4.1242	4.2759
#3	6.6901	6.2392	8.5470	2.6270	8.8712	3.8236	9.0094	3.1585
#4	6.2320	5.3751	5.7754	4.1733	5.6426	2.8426	6.3650	3.8355
#5	5.1357	4.9055	4.4046	2.8305	6.1027	3.4653	8.7862	3.6439

Table S4 List of the MTR_{mfit} quantified NOE(-1.6ppm) values from contralateral normal tissues (N) and stroke lesions (L) of five rat brains.

rat	Before		0.5-1h		1-1.5h		1.5-2h	
	N (%)	L (%)	N (%)	L (%)	N (%)	L (%)	N (%)	L (%)
#1	5.6516	5.4708	5.0105	1.5197	4.7570	3.0599	4.2765	2.4268
#2	5.8976	6.1588	3.4946	1.3598	3.3546	2.5424	1.8474	1.4289
#3	6.6316	6.1780	6.2197	1.9856	6.4453	1.8135	7.2943	1.9299
#4	5.2120	6.1923	5.1221	2.3426	5.2353	2.6077	5.2636	2.5449
#5	4.9988	5.2688	4.7076	2.3008	4.7849	3.0295	4.6705	2.3734

Table S5 List of the $AREX_{mfit}$ quantified NOE(-1.6ppm) values from contralateral normal tissues (N) and stroke lesions (L) of five rat brains.

rat	before		0.5-1h		1-1.5h		1.5-2h	
	N ($\%s^{-1}$)	L ($\%s^{-1}$)	N ($\%s^{-1}$)	L ($\%s^{-1}$)	N ($\%s^{-1}$)	L ($\%s^{-1}$)	N ($\%s^{-1}$)	L ($\%s^{-1}$)
#1	11.7318	11.0889	10.8396	2.7854	9.8489	5.3763	8.9555	4.2176
#2	12.2142	12.9690	7.0710	2.7164	6.6983	4.8820	3.6907	2.8181
#3	13.3058	11.9424	11.7107	3.1492	11.6613	2.7555	12.7897	2.6304
#4	9.6421	11.6351	9.8407	4.2419	10.0760	4.5252	9.6417	4.1503
#5	9.9817	10.6266	9.1227	3.9599	9.0551	5.0582	9.1831	3.7988

Table S6 List of the MTR_{mfit} quantified guanidine/amine CEST values from contralateral normal tissues (N) and stroke lesions (L) of five rat brains.

rat	Before		0.5-1h		1-1.5h		1.5-2h	
	N (%)	L (%)	N (%)	L (%)	N (%)	L (%)	N (%)	L (%)
#1	8.2793	8.2849	8.9516	7.4999	7.9107	7.9547	8.8106	8.3691
#2	8.5635	8.7923	7.6542	7.8645	9.6638	7.5819	9.3157	7.1344
#3	10.6927	8.8316	7.2670	7.6038	7.9177	7.1371	7.4823	6.5585
#4	7.4073	9.2840	8.2407	8.1244	9.9746	8.3703	8.6749	7.8692
#5	9.4164	10.2874	8.8887	7.9679	9.9024	9.2466	9.5776	9.8202

Table S7 List of the $AREX_{mfit}$ quantified guanidine/amine CEST values from contralateral normal tissues (N) and stroke lesions (L) of five rat brains.

rat	before		0.5-1h		1-1.5h		1.5-2h	
	N ($\%s^{-1}$)	L ($\%s^{-1}$)	N ($\%s^{-1}$)	L ($\%s^{-1}$)	N ($\%s^{-1}$)	L ($\%s^{-1}$)	N ($\%s^{-1}$)	L ($\%s^{-1}$)
#1	12.9004	12.9818	14.2296	10.7279	12.3074	11.0802	13.4466	11.3049
#2	13.7591	14.2566	11.7845	12.9855	14.1711	11.9269	13.9424	11.4419
#3	16.1952	13.2208	10.7648	9.8397	10.9611	8.6544	10.0361	7.4952
#4	11.0018	13.5625	12.4677	11.5579	14.7372	11.7398	12.3130	10.2182
#5	14.4782	16.0447	13.4531	11.0842	14.2237	12.1021	14.1853	11.7527

Table S8 List of the MTR_{mfit} quantified NOE(-3.5ppm) values from contralateral normal tissues (N) and stroke lesions (L) of five rat brains.

rat	Before		0.5-1h		1-1.5h		1.5-2h	
	N (%)	L (%)	N (%)	L (%)	N (%)	L (%)	N (%)	L (%)
#1	12.1568	11.5758	14.0636	12.5536	12.5788	12.6907	13.7818	13.8584
#2	12.2649	12.6073	12.9314	13.5644	15.2249	13.3311	15.1591	13.8961
#3	12.8602	13.0971	11.0818	14.4272	12.0699	13.4322	12.0331	13.4038
#4	11.1555	11.9701	11.7453	14.6616	13.9130	13.1647	12.5548	13.4864
#5	12.6028	12.9644	12.0541	12.9038	13.6006	13.8959	13.2450	16.2920

Table S9 List of the $AREX_{mfit}$ quantified NOE(-3.5ppm) values from contralateral normal tissues (N) and stroke lesions (L) of five rat brains.

rat	before		0.5-1h		1-1.5h		1.5-2h	
	N ($\%s^{-1}$)	L ($\%s^{-1}$)	N ($\%s^{-1}$)	L ($\%s^{-1}$)	N ($\%s^{-1}$)	L ($\%s^{-1}$)	N ($\%s^{-1}$)	L ($\%s^{-1}$)
#1	18.1072	17.1168	21.4040	16.4227	18.7791	16.4492	20.2888	17.3801
#2	17.6499	19.0965	18.0745	19.4198	20.7942	19.0451	21.0888	19.8909
#3	18.5695	18.1118	15.3769	16.3735	15.6198	14.4976	15.3314	13.5415
#4	15.2751	16.2844	16.5766	18.9967	19.2172	16.9040	16.6906	15.9091
#5	18.3017	18.9289	17.1267	16.4802	18.4410	17.1362	18.7544	18.3583

Table S10 List of the MTR_{mfit} quantified MT values from contralateral normal tissues (N) and stroke lesions (L) of five rat brains.

rat	Before		0.5-1h		1-1.5h		1.5-2h	
	N (%)	L (%)	N (%)	L (%)	N (%)	L (%)	N (%)	L (%)
#1	26.9468	27.1442	25.0356	26.4149	26.5797	26.4073	25.4476	25.0254
#2	26.2680	27.4631	25.1481	26.0611	22.6552	26.8294	23.8515	26.2697
#3	25.4609	25.0514	25.5352	24.9620	23.4345	25.2393	23.9914	24.2634
#4	25.6955	24.6164	25.4338	25.6249	23.4578	26.5736	24.5935	25.4647
#5	26.5602	26.7576	25.8517	26.3369	24.6760	25.3852	25.1501	21.1488

Table S11 List of the $AREX_{mfit}$ quantified MT values from contralateral normal tissues (N) and stroke lesions (L) of five rat brains.

rat	before		0.5-1h		1-1.5h		1.5-2h	
	N ($\%s^{-1}$)	L ($\%s^{-1}$)	N ($\%s^{-1}$)	L ($\%s^{-1}$)	N ($\%s^{-1}$)	L ($\%s^{-1}$)	N ($\%s^{-1}$)	L ($\%s^{-1}$)
#1	20.4054	20.3605	19.2050	17.9294	20.1348	17.4829	18.8556	15.9737
#2	19.6015	20.7833	18.3888	19.1485	16.0915	19.1105	16.9074	19.0114
#3	18.3139	17.7341	18.7402	14.3618	16.3278	14.2041	16.3226	12.9672
#4	18.5153	17.4512	18.7248	16.45382	16.6903	17.2429	16.9792	15.3521
#5	19.1701	19.3105	19.1129	17.3165	17.0356	15.8645	18.3785	12.4884

Table S12 List of T_{1w} values from contralateral normal tissues (N) and stroke lesions (L) of five rat brains.

rat	Before		0.5-1h		1-1.5h		1.5-2h	
	**N (s)**	**L (s)**	**N (s)**	**L (s)**	**N (s)**	**L (s)**	**N (s)**	**L (s)**
**#1**	1.8163	1.8393	1.7460	2.0124	1.8028	2.0625	1.8158	2.0961
**#2**	1.8212	1.8296	1.8316	1.8466	1.8294	1.9242	1.8595	1.8840
**#3**	1.8720	1.8986	1.8375	2.3366	1.8794	2.3928	1.9453	2.4901
**#4**	1.8763	1.8841	1.8297	2.1041	1.8435	2.1049	1.9300	2.2319
**#5**	1.8929	1.9009	1.8278	2.0717	1.9281	2.1555	1.8367	2.1720

Table S13 List of ADC values from contralateral normal tissues (N) and stroke lesions (L) of five rat brains.

rat	Before		0.5-1h		1-1.5h		1.5-2h	
	**N ($\mu m^2/ms$)**	**L ($\mu m^2/ms$)**	**N ($\mu m^2/ms$)**	**L ($\mu m^2/ms$)**	**N ($\mu m^2/ms$)**	**L ($\mu m^2/ms$)**	**N ($\mu m^2/ms$)**	**L ($\mu m^2/ms$)**
**#1**	0.6517	0.7000	0.6512	0.6225	0.6240	0.5936	0.6353	0.6028
**#2**	0.5968	0.6582	0.6454	0.4586	0.6461	0.4540	0.6847	0.4845
**#3**	0.6106	0.6740	0.7468	0.5805	0.6546	0.4039	0.7138	0.4659
**#4**	0.6095	0.6497	0.5875	0.4673	0.6214	0.5379	0.7134	0.5595
**#5**	0.5905	0.6425	0.6431	0.5809	0.6471	0.5801	0.6570	0.5836

Table S14 List of the MTR_{asym} quantified APT values from contralateral normal tissues (N) and stroke lesions (L) of five rat brains.

rat	before		0.5-1h		1-1.5h		1.5-2h	
	N (%)	L (%)	N (%)	L (%)	N (%)	L (%)	N (%)	L (%)
#1	-6.8093	-6.7570	-7.5666	-7.1103	-7.9032	-8.2428	-7.5777	-8.4852
#2	-5.2840	-6.7152	-6.3905	-8.4265	-6.1308	-8.5040	-6.8578	-9.0316
#3	-6.5217	-6.3574	-7.0276	-8.9401	-6.1449	-8.5629	-6.0679	-8.5953
#4	-6.6245	-6.2028	-6.7826	-8.9848	-6.3317	-7.7882	-6.2551	-7.9144
#5	-8.1315	-7.7891	-6.7430	-7.2029	-6.8496	-7.7144	-7.5785	-7.8258

Table S15 List of the $AREX_{asym}$ quantified APT values from contralateral normal tissues (N) and stroke lesions (L) of five rat brains.

rat	before		0.5-1h		1-1.5h		1.5-2h	
	N ($\%s^{-1}$)	L ($\%s^{-1}$)	N ($\%s^{-1}$)	L ($\%s^{-1}$)	N ($\%s^{-1}$)	L ($\%s^{-1}$)	N ($\%s^{-1}$)	L ($\%s^{-1}$)
#1	-10.9718	-10.7983	-12.7530	-10.2248	-12.6316	-11.4265	-12.1999	-11.5542
#2	-8.3585	-11.1138	-9.8739	-13.1583	-9.5703	-13.0677	-10.8222	-13.9507
#3	-10.3354	-9.8652	-10.4493	-11.0383	-8.7219	-10.0081	-8.4445	-9.3172
#4	-9.9535	-9.3653	-10.3340	-12.6135	-9.7269	-10.7405	-9.0958	-10.1142
#5	-12.9570	-12.5670	-10.3784	-9.9714	-10.3185	-10.5032	-11.7310	-10.0286

Table S16 List of the MTR_{LD_asym} quantified NOE(-1.6ppm) values from contralateral normal tissues (N) and stroke lesions (L) of five rat brains.

rat	before		0.5-1h		1-1.5h		1.5-2h	
	N (%)	L (%)	N (%)	L (%)	N (%)	L (%)	N (%)	L (%)
#1	3.1769	2.2560	3.1094	0.6189	2.6080	0.3738	2.4702	-0.2629
#2	4.9093	3.7956	2.7180	0.4065	2.3881	0.9617	0.5848	1.4507
#3	1.7057	2.3841	3.2184	0.5932	3.7990	1.5700	3.8736	1.2331
#4	2.5350	1.9602	1.6519	0.5685	1.7864	0.1928	2.4655	1.1250
#5	1.3343	1.2221	0.9821	0.5463	1.6635	0.5280	2.3703	0.7955

Table S17 List of the $AREX_{LD\_asym}$ quantified NOE(-1.6ppm) values from contralateral normal tissues (N) and stroke lesions (L) of five rat brains.

rat	before		0.5-1h		1-1.5h		1.5-2h	
	N ($\%s^{-1}$)	L ($\%s^{-1}$)	N ($\%s^{-1}$)	L ($\%s^{-1}$)	N ($\%s^{-1}$)	L ($\%s^{-1}$)	N ($\%s^{-1}$)	L ($\%s^{-1}$)
#1	7.4777	5.3598	7.9020	1.6549	6.6681	1.4342	6.5791	0.4266
#2	10.8342	8.8601	6.1281	1.1454	5.3659	2.3739	1.7736	3.1326
#3	4.3773	5.1011	6.6698	1.3225	7.3706	2.6271	7.3109	1.9592
#4	5.1054	4.2070	3.8137	1.6628	4.0573	0.8517	4.9980	2.2803
#5	3.3130	3.1713	2.5094	1.3698	3.9108	1.5076	5.6899	1.7757

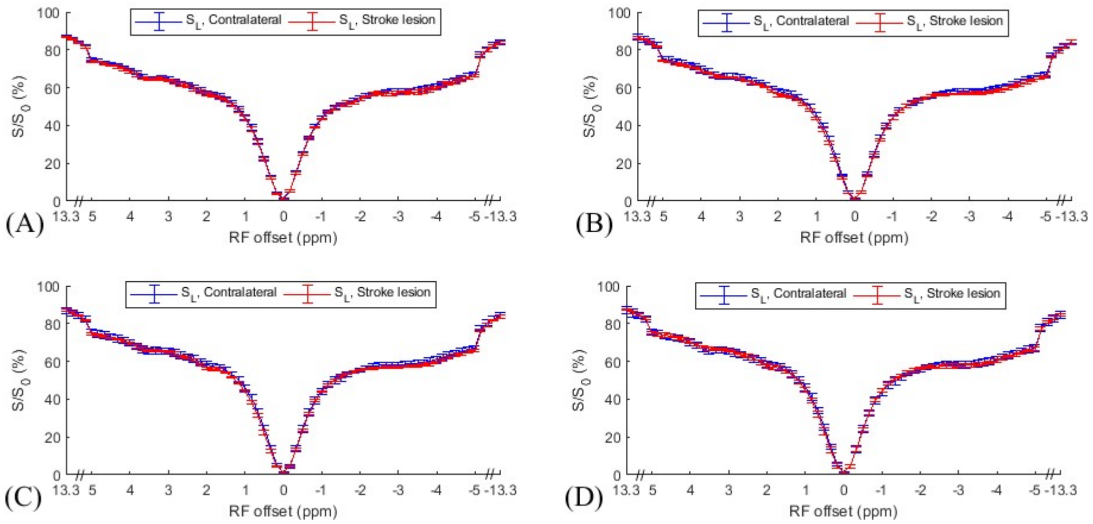

Figure. S1. Average and standard deviation of CEST Z-spectra (S_L) from stroke lesion (red) and contralateral normal tissues (blue) acquired before (A), 0.5-1 h (B), 1-1.5 h (C), and 1.5-2 h (D) after the onset of stroke.

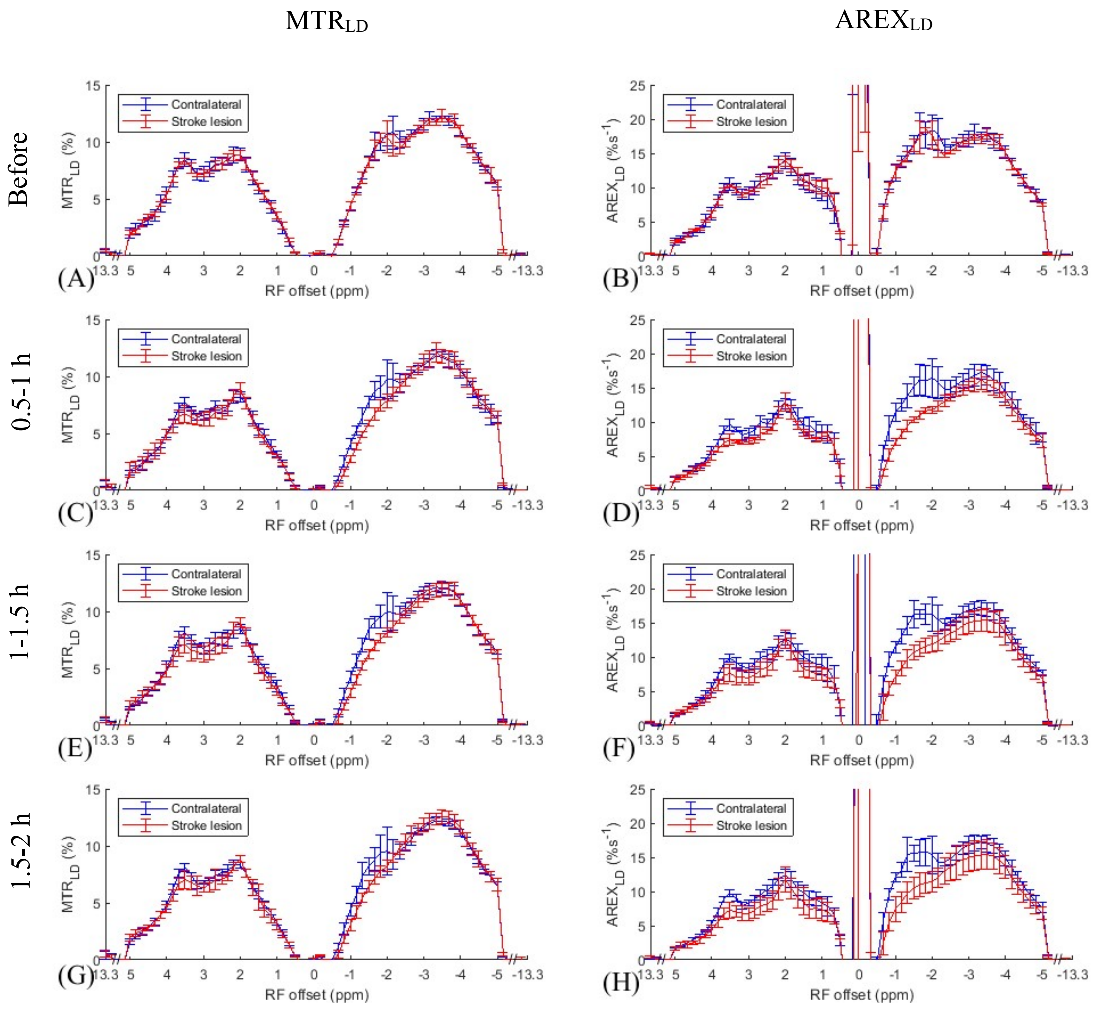

Figure S2. Average and standard deviation of MTR_{LD} spectra (left column) and $AREX_{LD}$ spectra (right column) from stroke lesion (red) and contralateral normal tissue (blue), respectively, acquired before (A, B), 0.5-1 h (C, D), 1-1.5 h (E, F), and 1.5-2 h (G, H) after the onset of stroke.

h

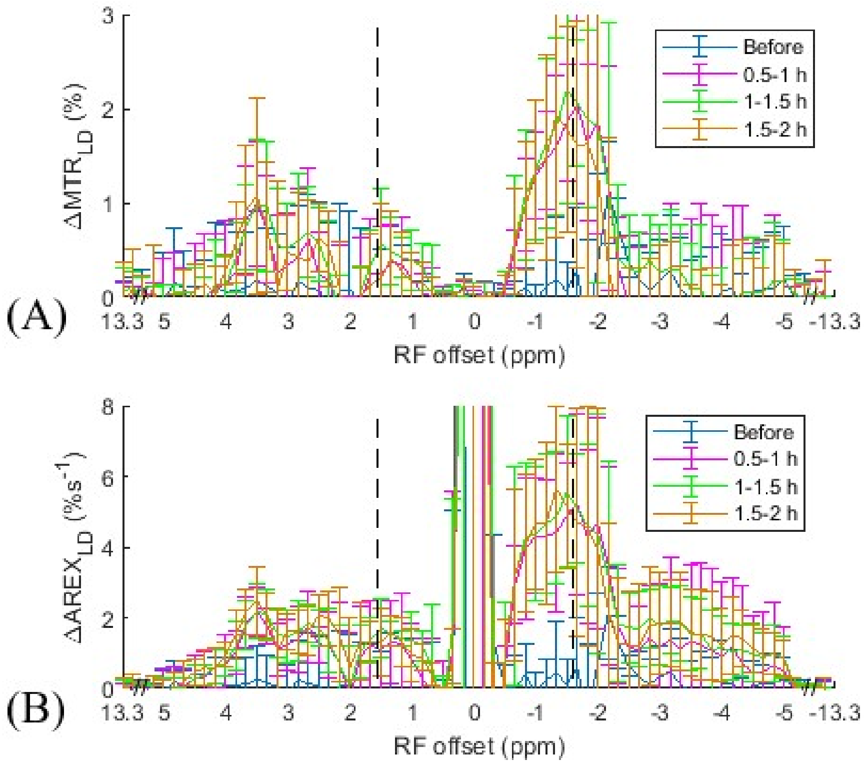

Figure S3. Average and standard deviation of ΔMTR_{LD} spectra (A) and $\Delta AREX_{LD}$ spectra (B) acquired before, 0.5-1 h, 1-1.5 h, and 1.5-2 h after the onset of stroke. Dashed lines show the RF frequency offsets at 1.6ppm and -1.6ppm, respectively.

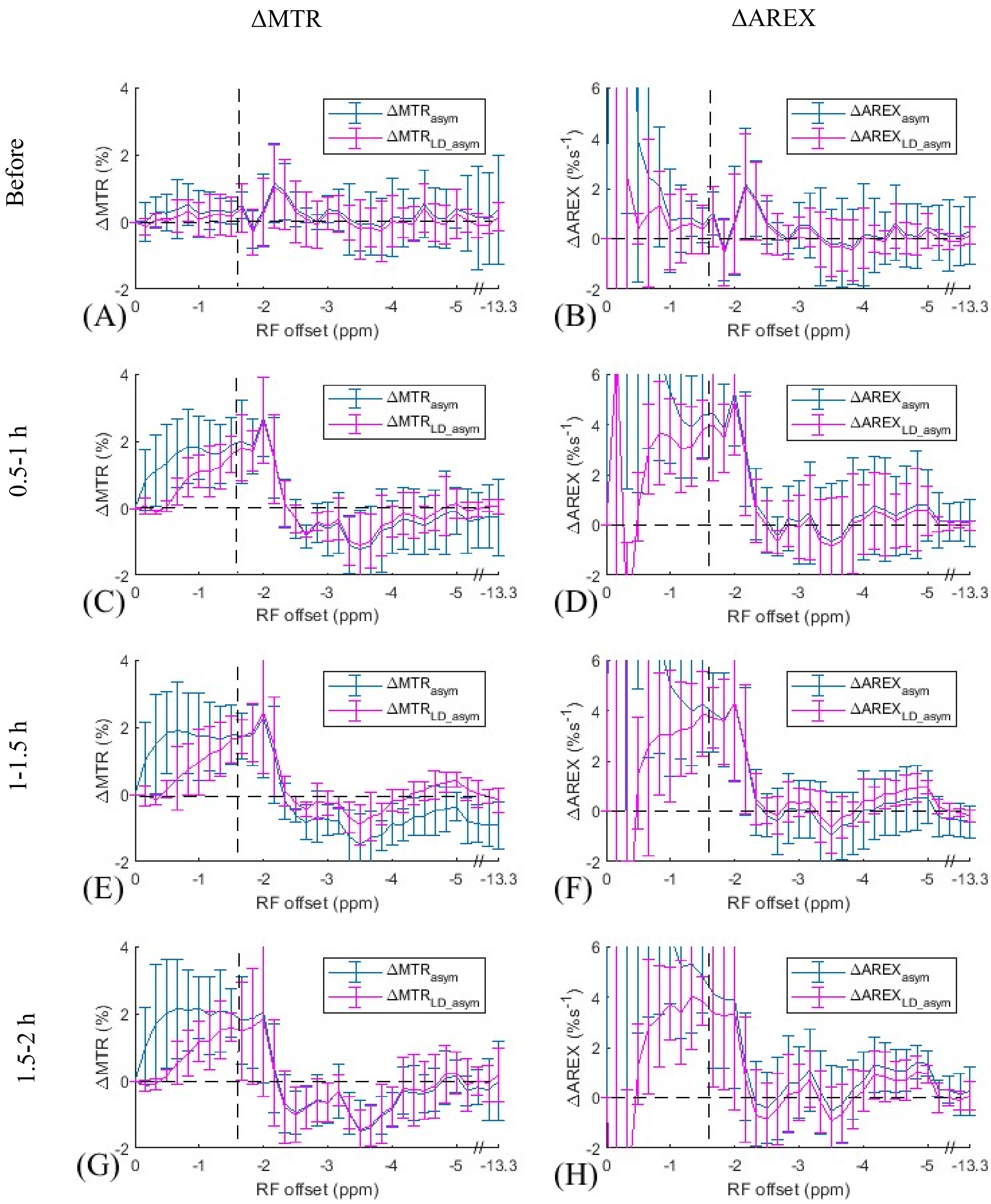

Figure S4. Comparison between the average and standard deviation of ΔMTR_{asym} and ΔMTR_{LD_asym} spectra (left column), as well as between the average and standard deviation of $\Delta AREX_{asym}$ and $\Delta AREX_{LD\_asym}$ spectra (right column), respectively, acquired before (A, B), 0.5-1 h (C, D), 1-1.5 h (E, F), and 1.5-2 h (G, H) after the onset of stroke. Dashed lines represent the ΔMTR values of 0% or ΔAREX values of $0\%s^{-1}$ and RF frequency offsets at -1.6ppm.

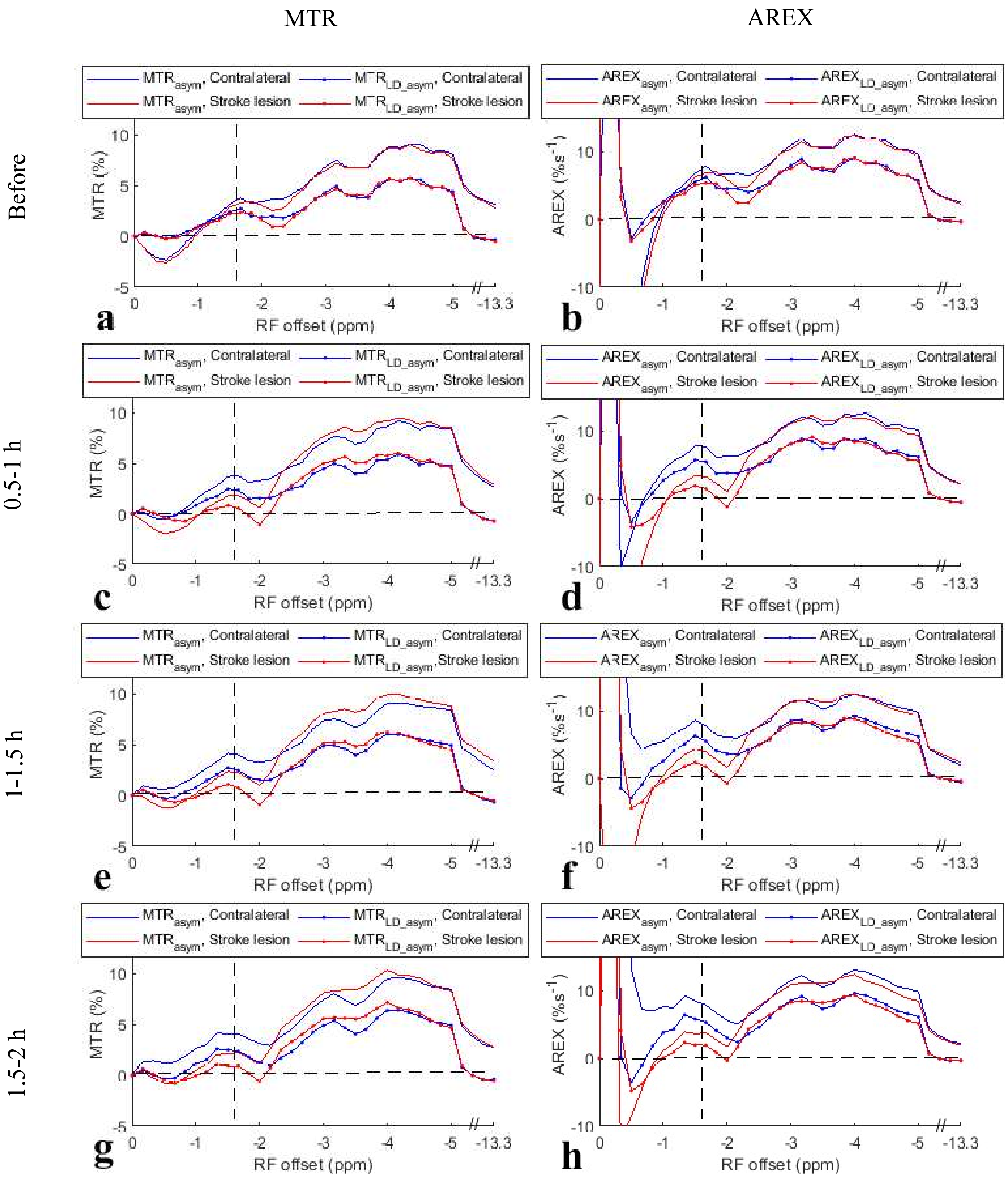

Figure S5. Comparison of the average MTR_{asym} and $AREX_{asym}$ spectra with the average MTR_{LD_asym} and $AREX_{LD\_asym}$ spectra from stroke lesion (red) and contralateral normal tissue (blue) of five rat brains, respectively, acquired before (A, B), 0.5-1 h (C, D), 1-1.5 h (E, F), and 1.5-2 h (G, H) after the onset of stroke. Dashed lines represent the MTR_{asym} values of 0% or $AREX_{asym}$ values of 0%s^{-1} and RF frequency offsets at -1.6 ppm.

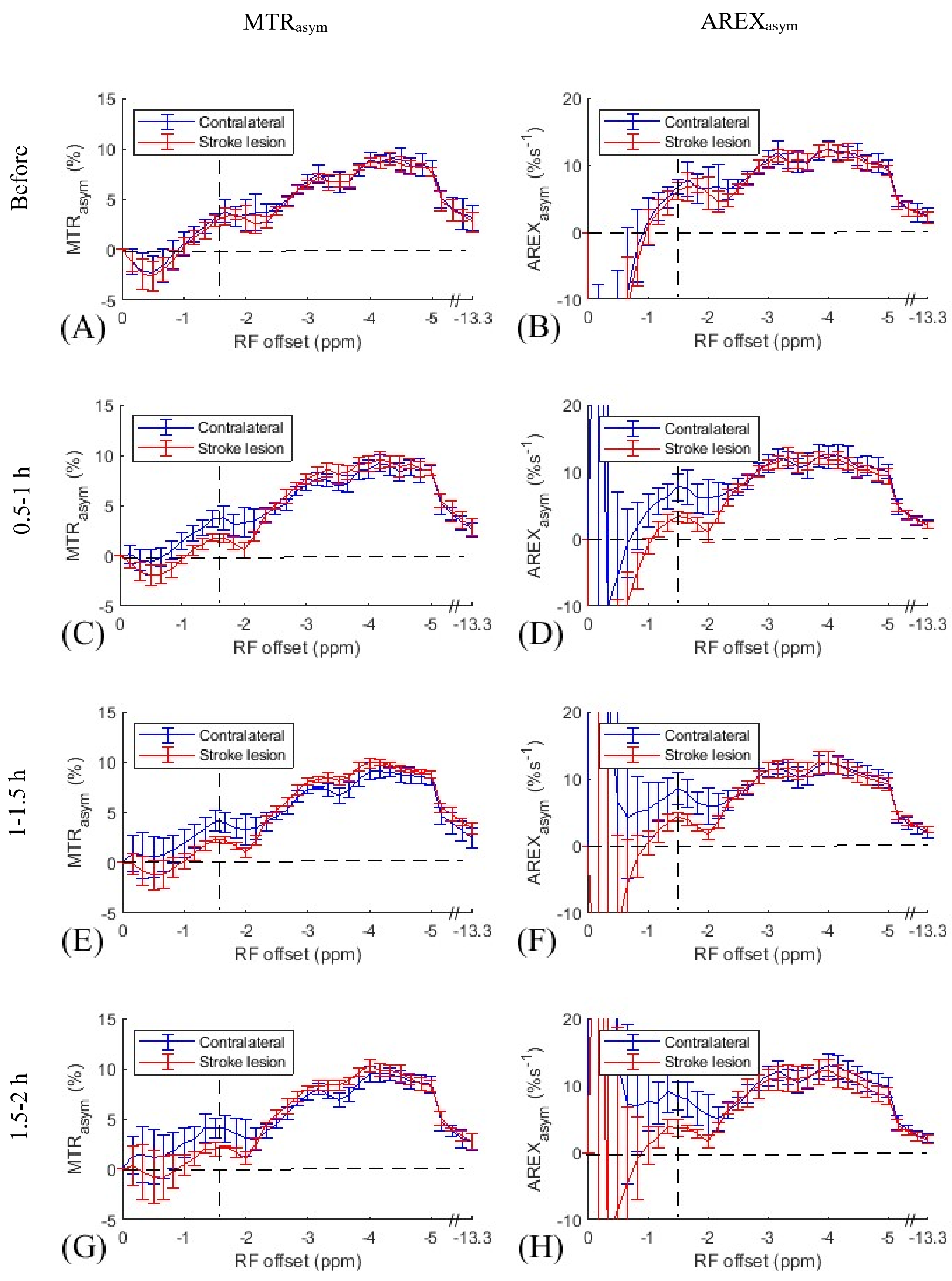

Figure S6. Average and standard deviation of MTR_{asym} spectra (left column) and $AREX_{asym}$ spectra (right column) from stroke lesion (red) and contralateral normal tissue (blue), respectively, acquired before (A, B), 0.5-1 h (C, D), 1-1.5 h (E, F), and 1.5-2 h (G, H) after the

onset of stroke. Dashed lines represent the MTR_{asym} values of 0% or $AREX_{asym}$ values of 0%s^{-1} and RF frequency offsets at -1.6ppm.

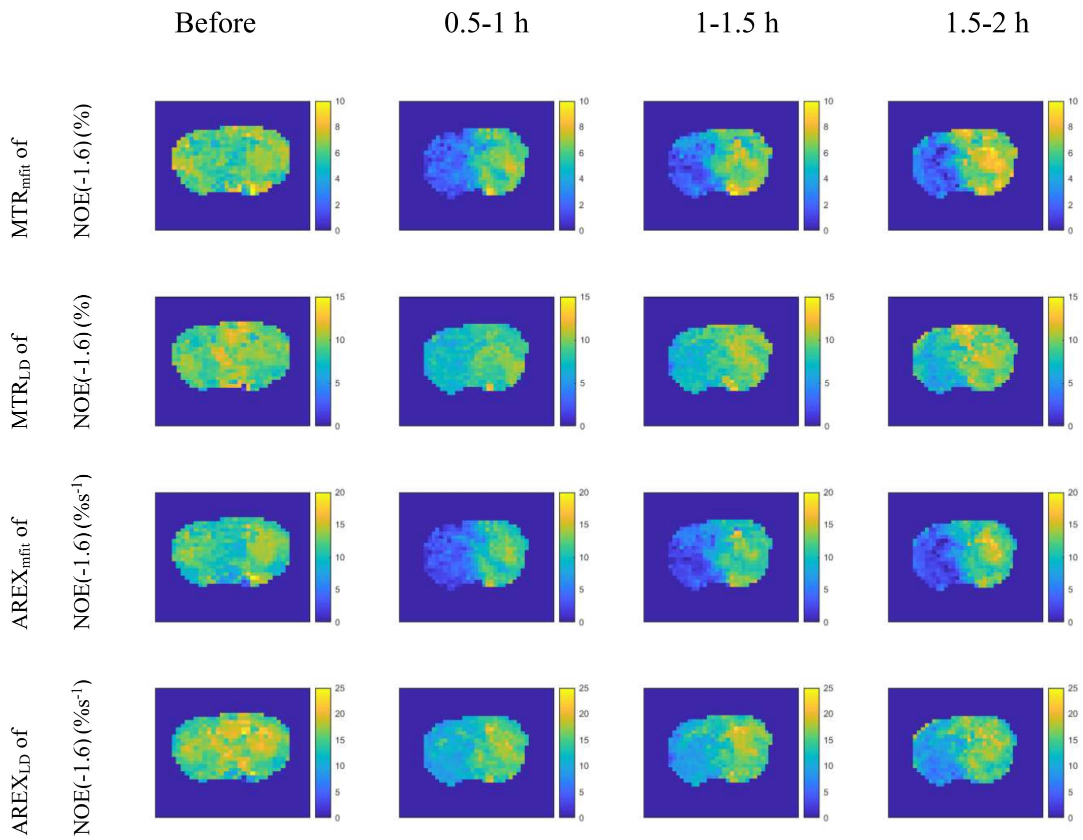

Figure S7. Maps of MTR_{mfit}, MTR_{LD}, $AREX_{mfit}$, and $AREX_{LD}$ quantified NOE(-1.6ppm) acquired before and at different time points after ischemic stroke from the brain of a representative rat.

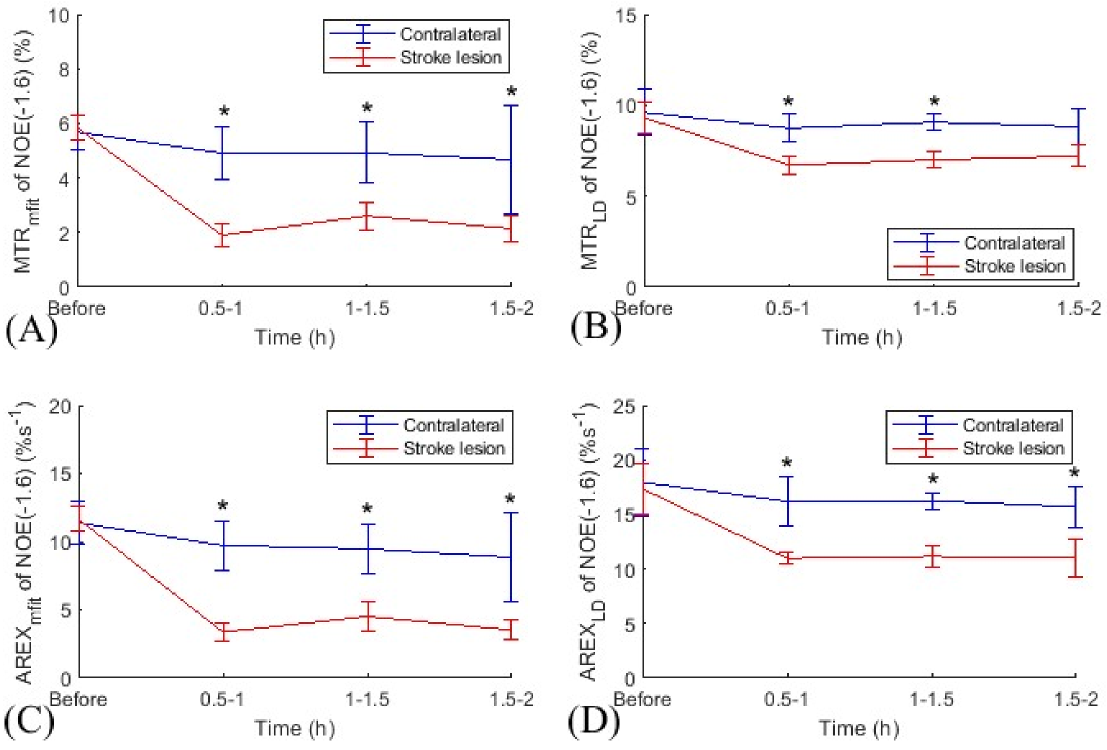

Figure S8. Time-dependent statistics of MTR_{mfit} quantified NOE(-1.6) (A), MTR_{LD} quantified NOE(-1.6) (B), $AREX_{mfit}$ quantified NOE(-1.6) (C), and $AREX_{LD}$ quantified NOE(-1.6) (D) in stroke lesions (red) and contralateral normal tissues (blue) of five rats. (* $P < 0.05$)

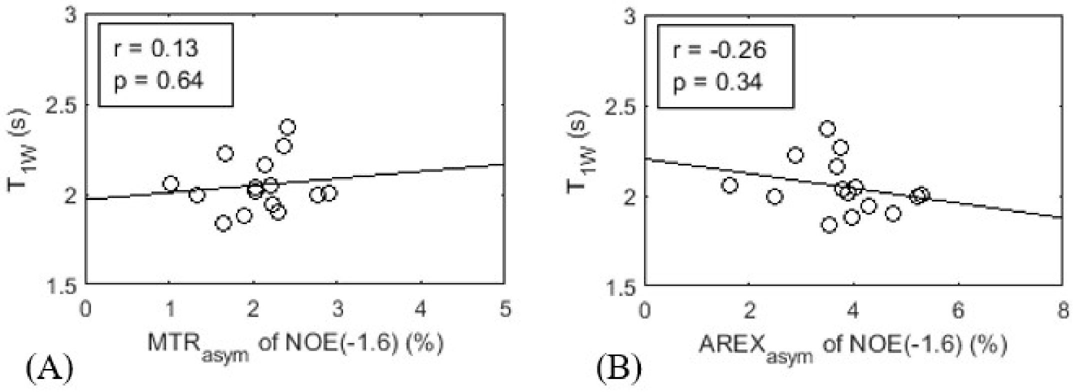

Figure S9. Summarized correlations of T_{1W} with MTR_{asym} quantified NOE(-1.6) (A) and $AREX_{asym}$ quantified NOE(-1.6) (B), respectively. The circles represent the mean values of each ROI from stroke lesion acquired at different time points after onset of stroke. Spearman's rank correlation coefficient (r) and p value of the correlation are provided. The solid line represents the linear regression of all data points.

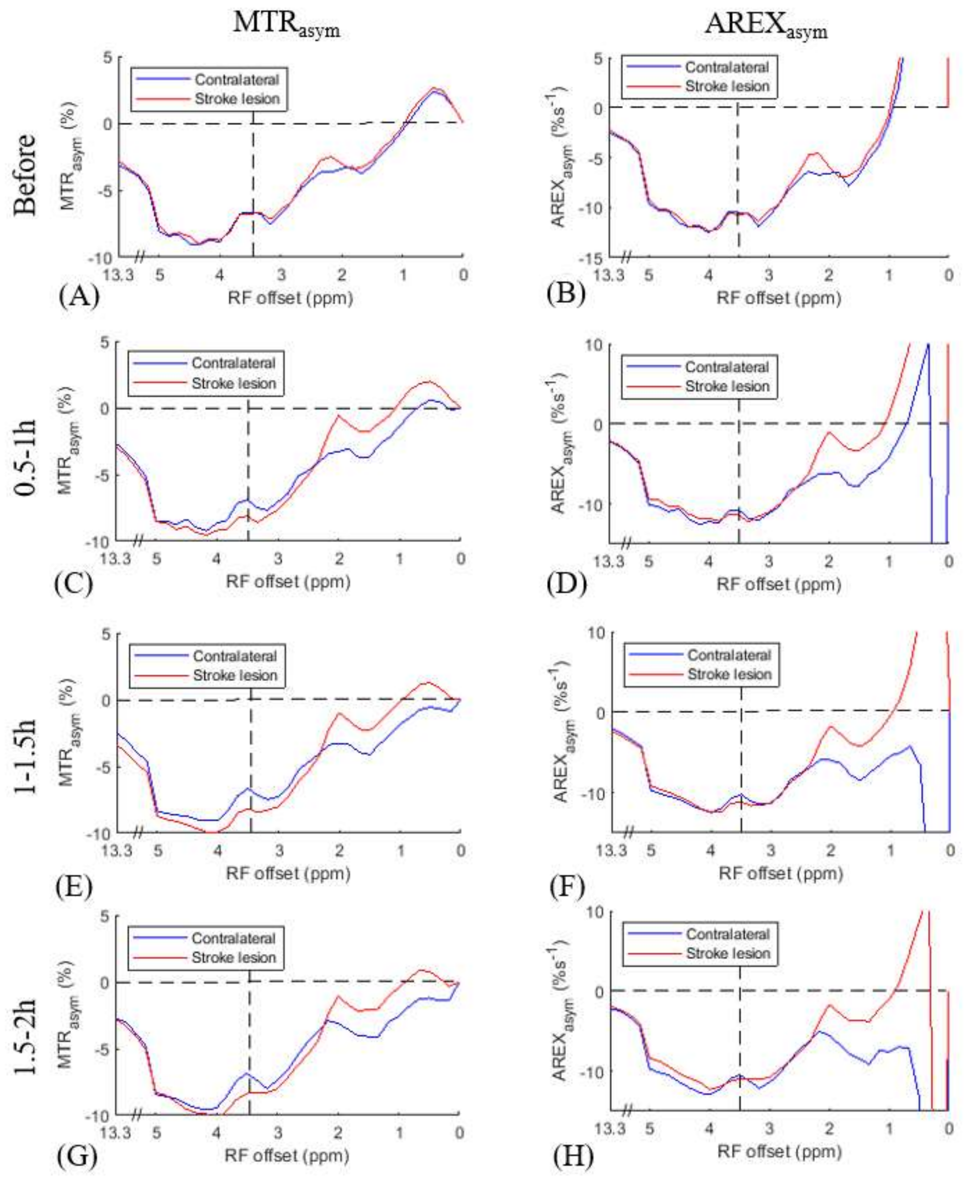

Figure S10. Average MTR_{asym} spectra (left column) and $AREX_{asym}$ spectra (right column) from stroke lesion (red) and contralateral normal tissue (blue) of the brains of 5 rats, respectively, acquired before (A, B), 0.5-1 h (C, D), 1-1.5 h (E, F), and 1.5-2 h (G, H) after the onset of stroke. Dashed lines represent the MTR_{asym} values of 0% or $AREX_{asym}$ values of 0%s^{-1} and RF frequency offsets at -1.6 ppm.

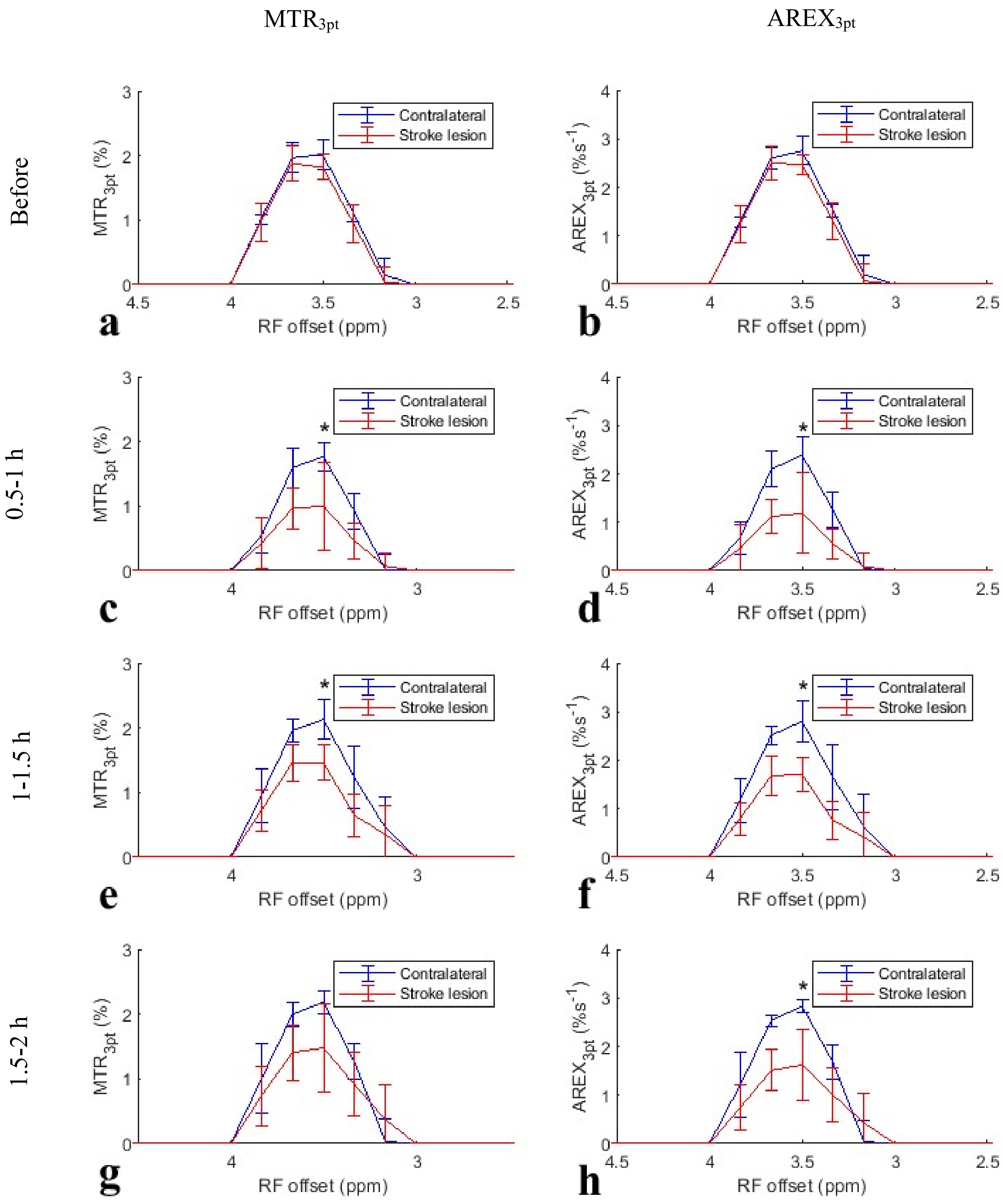

Figure S11. Average and standard deviation of MTR_{3pt} and $AREX_{3pt}$ quantified APT from stroke lesion (red) and contralateral normal tissue (blue), respectively, acquired before (A, B), 0.5-1 h (C, D), 1-1.5 h (E, F), and 1.5-2 h (G, H) after the onset of stroke. The three-point (3pt) quantification method uses the subtraction of a CEST signal acquired with RF saturation pulses at the resonance frequency of amide (3.5ppm) and an average of two CEST signals acquired at two nearby frequency offsets (3ppm and 4ppm). (* $P < 0.05$)
